# Core–Periphery Microbiota Organization Is Associated with Functional Resilience in High Arctic Svalbard Soils

**DOI:** 10.34133/csbj.0135

**Published:** 2026-06-24

**Authors:** Paweena Ouying, Suchana Chavanich, Voranop Viyakarn, Niranjan Divakaran, Naraporn Somboonna

**Affiliations:** ^1^ Biological Sciences Program, Faculty of Science, Chulalongkorn University, Bangkok 10330, Thailand.; ^2^ Reef Biology Research Group, Department of Marine Science, Faculty of Science, Chulalongkorn University, Bangkok 10330, Thailand.; ^3^ Aquatic Resources Research Institute, Chulalongkorn University, Bangkok 10330, Thailand.; ^4^ Department of Microbiology, Faculty of Science, Chulalongkorn University, Bangkok 10330, Thailand.; ^5^ Multi-Omics for Functional Products in Food, Cosmetics and Animals Research Unit, Chulalongkorn University, Bangkok 10330, Thailand.

## Abstract

**Experimental Objective:** Arctic soils harbor diverse bacterial communities that regulate carbon and nutrient cycling under persistent coldness, low-nutrient availability, and episodic freeze–thaw. However, the extent to which bacterial community stability versus spatial specialization structures these communities remains unresolved. **Methods:** This study characterized bacterial assemblages (microbiota) across 6 geographically distinct Svalbard soil sites using 16S ribosomal RNA gene sequencing, integrated with alpha- and beta-diversity comparisons, taxonomic profiling, linear discriminant analysis effect size (LEfSe) candidate biomarkers, core microbiota analysis, co-occurrence network, and predictive functional inference. **Results:** Proteobacteria dominated across Svalbard soil sites, accompanied by Actinobacteria, Acidobacteria, and Bacteroidetes. Taxa compositions and beta-diversity revealed spatial structuring, with Wahlbergøya and Northernmost Island relatively more unique community configurations. Consistently, LEfSe identified candidate site-specific taxa, i.e., sulfur-oxidizing bacteria and Acidobacteria, suggesting local environmental selection. The conserved core phylotype analysis indicated microbiota patterns associated with localized geographics and unraveled 48-phylotype core microbiota spanning 5 phyla. Functional predictions demonstrated dual-functional structures: (a) conserved functions in genome maintenance, membrane transport, and central carbon and amino acid metabolisms and (b) site-specific functional enrichments linked to stress responses, redox balance, xenobiotic degradation, and utilization of recalcitrant substrates. Integration of core microbiota taxonomic, network, and functional analyses further revealed 4 mechanistic resilience strategies: genome integrity maintenance, membrane adaptation, oxidative stress alleviation, and flexible resource (i.e., recalcitrant substrates) utilization. These strategies highlighted how shared core taxa collectively translated into microbial adaptive process and persistence, across sites. **Conclusions:** These findings underpinned that High Arctic soil microbiota were structured consistently with functional plasticity that supported Arctic ecosystem processes.

## Graphical Abstract

**Figure:**
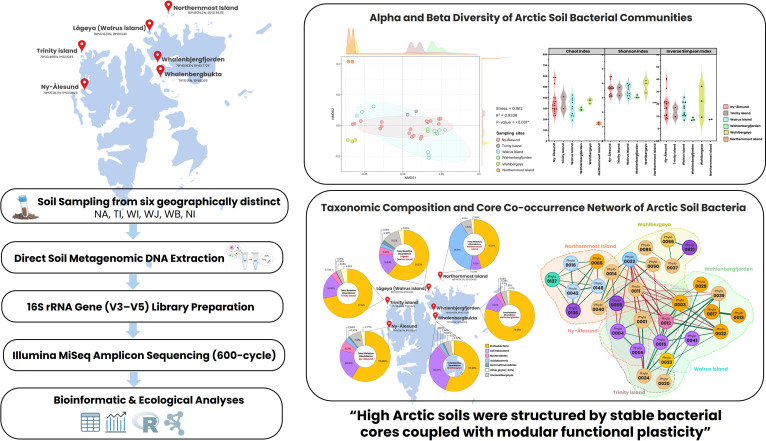

## Introduction

The Arctic, dominated by the ice-covered Arctic Ocean and bordered by the landmasses of Canada, Greenland, Finland, Iceland, Norway, Russia, Sweden, and Alaska, constitutes one of the most extreme biomes on Earth [1]. This region is characterized by persistently low temperatures, limited availability of liquid water, strong seasonality in solar and ultraviolet radiation, recurrent freeze–thaw cycles, episodic salinity fluctuations in coastal environments, and chronic nutrient limitation [2,3]. Collectively, these environmental stressors impose severe constraints on biological activity and microbial ecosystem functioning [4–6]. Svalbard, a High Arctic Norwegian archipelago situated between mainland Norway and the North Pole, represents a natural laboratory for polar microbiology. Ny-Ålesund, located on Spitsbergen, serves as a key year-round international research station for studies of Arctic ecosystems and climate change [6,7]. Investigations of cryospheric habitats, glaciers, snowpacks, and ice-marginal soils consistently identify bacteria as the dominant biota, where they mediate essential nutrient transformations and exhibit adaptations to chronic low temperatures (observed temperatures range from approximately −10 to 9 °C for this specific region), recurrent freeze–thaw cycles, oligotrophy, and pronounced seasonal variability [5,8–11]. Cold-adapted bacteria employ diverse physiological strategies, such as the production of ice-binding proteins reported in Gammaproteobacteria from Arctic sea ice and glacial environments, which enhance freeze tolerance and hold biotechnological applications in cryopreservation and food processing [12–15].

Arctic soils host diverse bacterial communities (microbiota) that play central roles in biogeochemical cycling and climate feedback regulation. Through persistent low temperatures, nutrient limitation, and local edaphic and climatic gradients, these communities exhibit taxonomic diversity and functional plasticity [16]. Recent work emphasized both the ecological breadth of Arctic soil microbiota and the need to better resolve their biogeographic distributions and functional attributes to improve predictions under rapid warming [17–19]. Arctic soils are dominated by Proteobacteria, Actinobacteria, and Acidobacteria, owing to soil pH, moisture, organic matter quality, vegetation cover, and disturbance history (i.e., wildfire, permafrost thaw, cryoturbation, glacial retreat, seabird nutrient deposition, and anthropogenic activities) characteristics [16,20–24]. Notably, Acidobacteria previously exhibited negative correlations with pH, whereas Proteobacteria tended to increase in higher-pH or carbon-enriched soils, reflecting broader oligotrophic–copiotrophic life-history strategies [4]. Yet, the extent to which these current taxonomic patterns translate into conserved functional capacities and reflect locally specialized adaptations remains unresolved, particularly the diversity and functional meaningful across the heterogeneous coastal and inland soils of Svalbard [16,17].

Culture-independent approach and high-throughput sequencing are well-accepted techniques to comprehensively characterize microbiota compositions, potentially core and co-occurrence taxa, and microbial functional potentials [25–28]. We thereby investigated spatial variation in soil microbiota across 6 locations in the Svalbard Archipelago (Ny-Ålesund, Trinity Island, Lågøya [Walrus Island], Wahlenbergfjorden, Wahlbergøya, and Northernmost Island) using 16S ribosomal RNA (rRNA) gene V3–V5 sequencing and comprehensive bioinformatic analyses. Specifically, we asked (a) how alpha- and beta-diversity and taxonomic compositions vary among sites, (b) which candidate site-associated taxa were for various exploratory sites, (c) whether an archipelago-wide core microbiota can be defined and how the taxa co-occur, and (d) which predicted microbial functions are conserved across sites versus locally enriched and how these functional profiles associate with core taxa. By integrating community profiling, biomarker analysis, core microbiota delineation, co-occurrence taxa inference, and functional prediction, this study provided a comprehensive assessment of soil bacterial biogeography across somewhat environmentally different sites in the Svalbard Archipelago. These insights served as a baseline survey of different sites across the archipelago for reference for future studies that evaluate how Svalbard has changed after climate change had drastically affected polar environments in the upcoming decades [29].

## Materials and Methods

### Soil sample collections and site description

**Fig. 1.**
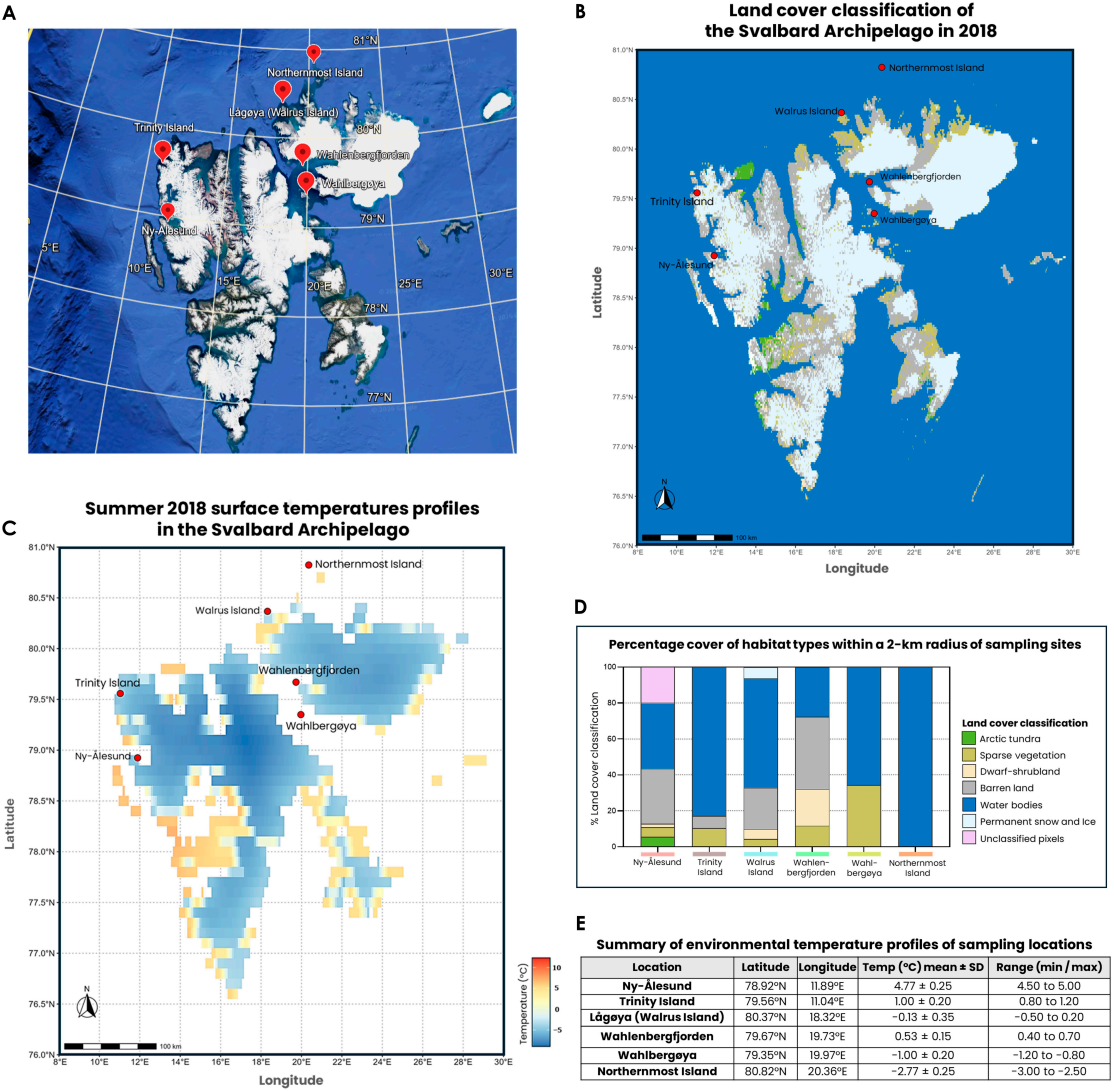
Geographical coordinate map and environmental parameters of 6 soil sampling sites in the High Arctic regions of the Svalbard Archipelago, Norway: Ny-Ålesund, Trinity Island, Lågøya (Walrus Island), Wahlenbergfjorden, Wahlbergøya, and Northernmost Island. (A) Spatial coordinates, (B) terrestrial land cover habitat classification, (C) mean surface temperature in the summer season of year 2018 based on ERA5-Land (European Centre for Medium-Range Weather Forecasts Reanalysis Fifth Generation-Land), (D) habitat types based on percent land cover classes, and (E) summary of Global Positioning System (GPS coordinates) and temperature profiles.

Soil samples were collected from 6 geographically different locations across the Svalbard Archipelago, the High Arctic region of Norway. The sampling sites included Ny-Ålesund (78°55′28.1″N, 11°53′28.2″E), Trinity Island (79°33.496′N, 11°02.104′E), Lågøya or Walrus Island (80°22.023′N, 18°19.124′E), Wahlenbergfjorden (79°40.163′N, 19°43.772′E), Wahlbergøya (79°21.01′N, 19°58.20′E), and Northernmost Island (80°49′24.2″N, 20°21′34.6″E) (Fig. 1A) [30]. These sites encompassed a range of environmental conditions (i.e., varying land cover classes and localized surface temperature gradients) [29,31,32], and soil samples were collected in the Arctic summer season between July and August 2018. Samples representing Ny-Ålesund (abbreviated NA) and Walrus island (WI) were collected along the shoreline and inland, while samples of other sites were collected only along the shoreline due to limited inland accessibility. Hence, NA and WI soil sites contain diverse soil structures, environmental conditions and habitat types, and 6 NA areas (NA1 to NA6) and 4 WI areas (WI1 to WI4) were selected (Table S1). Ny-Ålesund and Walrus Island include multiple sub-sites, correlating differences in microhabitats and environmental gradients within the sites: for site NA, the 6 areas attempted to capture the spatial heterogeneity of some soil composition, moisture availability, and vegetation cover effects, given that the biological replicates within each area were approximately 200 m apart (e.g., NA4_1, NA4_2, and NA4_3); for site WI, the areas WI1 and WI2 were located inland adjacent to a tidal pool, while WI3 and WI4 were located along the shoreline, and the biological replicates of each area were collected at approximately 100 m apart. Of each location, approximately 30 g sampling per sample was conducted at a depth ≤5 cm to represent the biologically active topsoil layer and 2 to 3 independent biological replicate samples were collected (e.g., NA1_3, NA1_2, and NA1_3 represent 3 independently replicate samplings of NA1; Northernmost Island NI_1 and NI_2 represent 2 independently replicate samplings of NI). Note that the biological replicates for each sampling site were as follows: NA (6 areas and *n* = 3 each), Trinity Island (abbreviated TI) (1 area and *n* = 2), WI (4 areas and *n* = 2 to 3 each), Wahlenbergfjorden (WJ) (1 area and *n* = 3), Wahlbergøya (WB) (1 area and *n* = 3), and Northernmost Island (NI) (1 area and *n* = 2), resulting in 38 total samples (Table S1). All collected samples were immediately preserved in a −80 °C portable freezer during transport.

For site description, environmental parameters included land cover types using the European Union’s Copernicus Global Land Cover database [33] and thermal profiles at a 2-m height using ERA5 (European Centre for Medium-Range Weather Forecasts Reanalysis Fifth Generation)-Land monthly averaged reanalysis on single levels [34], and habitat classification was analyzed using the numeric Land Cover Classification System codes that classified into 6 dominant Arctic categories: Arctic tundra, sparse vegetation, shrubland, barren land, water bodies, and permanent snow and ice. Note that the unclassified pixels at site NA inferred data gaps from topographic shading, cloud cover, or interference from anthropogenic infrastructure (Fig. 1B to E).

### Metagenomic DNA extraction

Metagenomic DNA was extracted from 250 mg of each sample, with 2 to 3 independent replicate extractions per sample, following the DNeasy PowerSoil DNA Isolation Kit (Qiagen, Hilden, Germany) and the manufacturer’s protocol. The quality and quantity of the metagenomic DNA were evaluated by 1% agarose gel electrophoresis and a NanoDrop spectrophotometer (Thermo Fisher Scientific, Massachusetts, USA) [35–39]. The metagenomic DNA was stored at −20 °C.

### 16S rRNA gene library preparation and high-throughput sequencing

The extracted DNA were polymerase chain reaction (PCR) amplified targeting the 16S rRNA gene hypervariable region V3–V5 using the universal primers 342F (5′-GGRGGCAGCAGTNGGGAA-3′) and 895R (5′-TGCGDCCGTACTCCCCA-3′) [39–41], which included Illumina MiSeq adapter sequences, linker regions, and unique 12-base pair barcodes for multiplexed sample identification. Each PCR comprised 25 μl containing 50 to 100 ng of the extracted DNA, 1× EmeraldAmp GT PCR Master Mix (TaKaRa Bio, Shiga, Japan), 0.2 μM of each primer, and nuclease-free water. The PCR conditions were 94 °C for 3 min, 25 cycles of 94 °C for 45 s, 50 °C for 1 min, and 72 °C for 1.5 min, followed by 72 °C for 10 min [42–44]. All samples were amplified in triplicate to minimize PCR bias. The amplicons were excised from agarose gels and purified using the GF-1 Gel Extraction Kit (Vivantis Technologies, Selangor, Malaysia), and the amplicon DNA was quantified using the Qubit dsDNA HS Assay Kit (Invitrogen, Oregon, USA) and Qubit 3.0 Fluorometer (Thermo Fisher Scientific). Then, the amplicons were pooled in equal concentrations (200 ng/sample) and sequenced using the Illumina MiSeq 600-cycle platform (Illumina, California, USA) at the Omics Sciences and Bioinformatics Center, Faculty of Science, Chulalongkorn University (Bangkok, Thailand) [39,42,43,45].

### Bioinformatics and statistical analyses

Sequencing data were processed following Mothur 1.47.0’s standard operating procedures for Illumina MiSeq [37,38,45–48]. Quality screening included removal of short reads <100 nucleotides, chimera sequences, and sequences with >8 nucleotide ambiguous bases or homopolymers >10 nucleotides. The quality reads were aligned with the SILVA database (release 138.1) to exclude nonbacterial sequences of mitochondria, chloroplasts, and unclassified lineages [49,50]. Operational taxonomic units (OTUs) were classified based on phylotype using a naïve Bayesian classifier into phylum, class, order, family, genus, and species levels [51]. Alpha-diversity analyses included Chao1 richness, Shannon diversity, inverse Simpson, and Good’s coverage (estimated sequencing depth of the sample relative to the true sample diversity), and beta-diversity analyses included Thetayc dissimilarity metric and nonmetric multidimensional scaling (NMDS), computed using Mothur 1.47.0 [37,38,46–48,52–56]. Taxonomic profiles at the phylum level were further analyzed by GraphPad Prism 10.2.3 [57]. Note that genera for which the genus names could not be identified were inferred by a small letter to the deepest taxonomic names that could be identified (c_ for class, o_ for order, f_ for family, and g_ for genus), and we further analyzed some unclassified genera following established methods by BLASTN *vs.* National Center for Biotechnology Information (NCBI) nonredundant database with a significant *E* value and percent sequence coverage >80% [38,58–60]. Estimated functions of the microbial communities were investigated using Phylogenetic Investigation of Communities by Reconstruction of Unobserved States (PICRUSt2), with functional categories annotated following the MetaCyc metabolic pathway database [61–64]. The principal component analysis was computed to infer microbial pathways clustering and visualized in R version 4.4.2 using the ggplot2 and ggExtra packages. The statistical comparisons of the predicted metabolic functions were performed using the STAMP software [65]. The nearest sequenced taxon index (NSTI) was included to evaluate the reliability of PICRUSt2 inference, and to account for the nonnormal distribution and compositional nature of the data, White’s nonparametric *t* test (for comparing microbial proportions) was employed. Multiple testing was corrected using the Benjamini–Hochberg false discovery rate (FDR), with the significance threshold set at *P* < 0.01 [66,67]. Differentially abundant taxa across sampling sites (estimated specific taxa biomarkers) were identified using linear discriminant analysis effect size (LEfSe) at the species level using Mothur 1.47.0 [46,47,68]. LEfSe was performed using relative abundance data derived from the same rarefied sequences per sample (rarefied OTU table) to allow consistency of normalization. Differential abundance testing was performed using the nonparametric Kruskal–Wallis and Wilcoxon rank-sum tests on relative abundance profiles, and statistical significance LEfSe was reported as logarithmic linear discriminant analysis (LDA) effect-size scores. Taxa were considered significant when they satisfied both *P* < 0.05 and LDA score > 2.4, and only the statistically significant putative taxa biomarkers were visualized using GraphPad Prism 10.2.3. Species-level assignments derived from the 16S rRNA gene V3–V5 regions were interpreted as best-match annotations, whereas genus-level classifications were considered more taxonomically reliable. LEfSe results were interpreted as exploratory candidate biomarkers rather than definitive indicators of differential abundance following the compositional nature of microbiota datasets.

Core microbiota (or core phylotypes) were defined in R version 4.4.2, and protocols were established on the following criteria: (a) presence in all samples, (b) the relative percent abundance is >0.1% in every individual sample, and (c) the average relative percent abundance across all samples is >1% [39,69,70]. This approach ensured the inclusion of consistent and abundant microbial taxa. Then, Spearman’s correlation was used to examine associations between the core phylotypes and the predicted MetaCyc pathways using the Hmisc package in R version 4.4.2. Correlation coefficients and corresponding *P* values were calculated using the rcorr function. To reduce weak or potentially spurious associations, only strong positive correlations meeting the thresholds of Spearman’s *ρ* > 0.7 and *P* < 0.05 were retained for visualization by ggplot2 and reshape2 [67,70,71]. The circular phylogenetic dendrogram of core phylotypes was computed based on Thetayc beta-diversity coefficients [46,47,72], and hierarchical taxonomy from phylum to species levels was illustrated by iTOL 7.2.1 [73]. To examine microbial co-occurrence patterns among conserved core phylotypes, co-occurrence networks were constructed using Sparse Correlations for Compositional data (SparCC), identifying significant correlations (FDR-adjusted *P* < 0.05) with a strong correlation threshold |*ρ*| > 0.6 [74,75]. Note that the network analysis was restricted to core OTUs to focus on consistently detected and abundant taxa across samples, and the inferred associations represent statistical co-occurrence relationships and should not be interpreted as direct ecological interactions. The resulting networks were visualized using Cytoscape 3.10.3 [76,77].

Statistical differences between samples (or groups) were assessed using the nonparametric Kruskal–Wallis *H* test. Post hoc pairwise comparisons were performed using Dunn’s test with *P* values adjusted for multiple comparisons using the Benjamini–Hochberg method [67,78–80]. Among samples (or groups), analysis of molecular variance (AMOVA) was conducted using Mothur 1.40.7 [81], and/or permutational multivariate analysis of variance (PERMANOVA) was conducted using the adonis2 function in the vegan R package [82,83]. To validate the PERMANOVA results, a test of homogeneity of multivariate dispersions (PERMDISP) was utilized to confer if the significant differences detected by PERMANOVA were attributed to shifts in community compositions rather than unequal group variances (dispersion) [84]. All statistical analyses and visualizations were executed in R version 4.4.2 [85–87]. A summary of the statistical analyses, normalization procedures, significance thresholds, and associated software/packages used in this study is provided in Table S2.

Moreover, in parallel to Mothur’s OTU microbiota analysis, amplicon sequence variants (ASVs) were also identified to ensure consistency between the microbiota analysis approaches [88]. Sequences were classified via the SILVA 138.1 database. Of alpha- and beta-diversity indices, the data were calculated based on normalized sequencing depths per sample. Taxonomic compositions and relative abundances were determined using the nonsubsampled (total) dataset to maintain the comprehensive microbial profile and minimize the exclusion of rare taxa [46,47,49,50].

## Results

### 16S rRNA gene sequences and alpha-diversity

The 16S rRNA gene sequences of samples NA1, NA2, NA3, NA4, NA5, NA6, TI, WI1, WI2, WI3, WI4, WJ, WB, and NI (each with independent 2 to 3 replicates) had average 65,039 raw reads and 58,939 quality reads, per sample, respectively (Table S1). To ensure uniform sequencing depth and comparability across samples, the data were rarefied to 11,320 quality reads per sample (corresponding to the lowest read count among the samples) as the baseline normalization for calculating taxonomic relative abundances and prior to any statistical comparisons, and the average Good’s coverage to estimate the sequencing coverage to the true microbial diversity at this rarefaction depth was found to be 99.97% ± 0.10% at the phylum classification level and 99.37% ± 0.21% at the genus classification level (Table S3a). At the genus level, Good’s coverage percentages ranged from 98.77% to 99.75%, consistent with the reaching plateau of the rarefaction curves (Fig. S1). Following the Good’s coverage and rarefaction curve results, this rarefied dataset still retained all samples for downstream comparative analyses for alpha-diversity, beta-diversity, taxonomic composition, and statistical community comparisons.

The alpha-diversity of bacterial communities in the Svalbard soil samples revealed averages of 19 phyla and 249 genera per site. Site NI exhibited the lowest genus richness (112 ± 4.24 genera), WJ, 207 ± 11.93 genera; WI, 236 ± 11.68 genera; TI, 265 ± 59.40 genera; and NA 272 ± 27.47 genera, to the highest at WB with 279 ± 34.22 genera, consistent with Chao1 richness (Table S3b and Fig. S2). Considering both richness and evenness components demonstrated that Shannon and inverse Simpson diversity indices differed significantly among sites (*P* = 0.04 for both), although post hoc pairwise comparisons were not statistically significant (*p_adj_* > 0.05) (Table S1S4). Ny-Ålesund and Wahlbergøya generally exhibited relatively high diversity and evenness (Fig. S2).

### Taxonomic distribution and beta-diversity of soil microbiota across the High Arctic sites

**Fig. 2.**
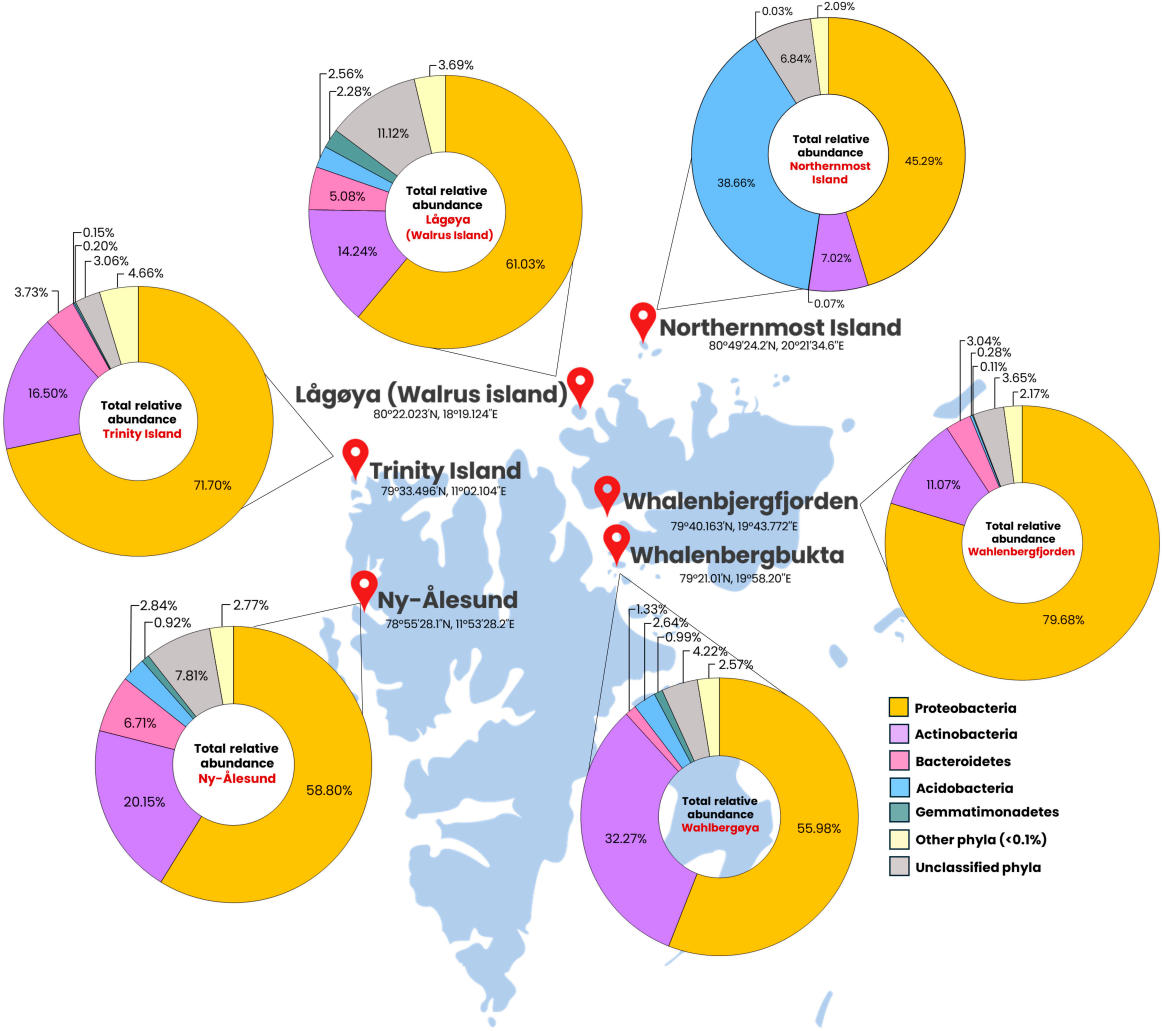
Pie chart showing the average percent relative abundances of bacterial phyla across all sampling sites. Bacterial phyla that had <0.1% were grouped under the category “Other phyla (<0.1%)”, and taxa that could not be confidently assigned to phyla were under “Unclassified phyla”.

The taxonomic profiles revealed Proteobacteria as the dominant phylum across all sites (avg. 62.17% ± 12.10%, lowest 45.29% in Northernmost Island and highest 79.68% in Wahlenbergfjorden), followed by Actinobacteria (avg. 16.74% ± 8.62%, lowest 7.02% in Northernmost Island and highest 32.27% in Wahlbergøya) and Bacteroidetes (avg. 3.35% ± 2.43%, lowest 0.10% in Northernmost Island and highest 6.71% in Ny-Ålesund) (Fig. 2). Acidobacteria represented the fourth most abundant phylum on average, but it was uniquely abundant in Northernmost Island (38.66%). The rather distinct profile in Northernmost Island agreed with its environmental conditions of a relatively lowest temperature of −2.77 ± 0.25 °C and a habitat type primarily of water bodies (Fig. 1D and E). Meanwhile, Gemmatimonadetes were detected across sites but remained below 10%, and unclassified phyla were on average 6.08% in the Svalbard (lowest 3.06% in Trinity Island and highest 11.12% in Walrus Island) (Fig. 2).

At the genus level, f_Rhodobacteraceae, c_Gammaproteobacteria, and f_Acetobacteraceae were relatively predominant in Proteobacteria; *Ilumatobacter*, f__C111, and f__JdFBGBact in Actinobacteria; and f__Flavobacteriaceae in Bacteroidetes (Fig. S3a). From a total of 982 bacterial genera across all sites, a heatmap of the top 20 most abundant genera was generated. Members of the phylum Proteobacteria were consistently predominant across all sites, for example, an unclassified genus in *Rhodobacteraceae* (particularly abundant in Ny-Ålesund, Trinity Island, Walrus Island, and Wahlenbergfjorden) and *Pseudomonas* (particularly abundant in Wahlbergøya). Different High Arctic sites demonstrated different composition trends, and an unclassified genus in family *Acetobacteraceae* was uniquely high in Northernmost Island soil (Fig. S3b and Table S5). Additionally, genera affiliated with the phylum Actinobacteria, including *Ilumatobacter*, f_C111 (BLASTN *vs.* NCBI nonredundant database with an *E* value of 0.00 as *Desertimonas*), and f_JdFBGBact (BLASTN *E* value 7e−166, *Actinomarinicola*), were primarily detected at Ny-Ålesund, Trinity Island, Walrus Island, and Wahlenbergfjorden, distinct from sites Wahlbergøya and Northernmost Island, where o_Actinomycetales (BLASTN *E* value 0.00, *Jatrophihabitans*) was primarily detected. Notably, sites Wahlbergøya (sparse vegetation), Ny-Ålesund (Arctic tundra and sparse vegetation), and TI showed higher vegetation cover percentages (Fig. 1D). Moreover, a genus among the top 20 abundant ones in phylum Bacteroidetes was relatively more prevalent at sites NA, TI, WI, and WJ than in WB and NI (f_Flavobacteriaceae), and in phylum Acidobacteria, f_Acidobacteriaceae (BLASTN *E* value 0.00, *Silvibacterium*) was primarily prominent in NI. Together, the heatmap clearly exhibited separate patterns in microbiota profiles (Fig. S3b and Table S5).

To evaluate the differences among bacterial genus communities across the 6 sites, NMDS analysis based on Thetayc dissimilarity metrics demonstrated significant separation among bacterial communities across the 6 sites (AMOVA and PERMANOVA, *P* ≤ 0.001; Fig. S4a and Table S6). The NMDS model showed acceptable ordination performance (stress = 0.15 and *R*^2^ = 0.93) [56,72]. The additionally significant PERMDISP test (*P* = 0.020) confirmed that the differences were due to site variations [81–84]. Along the NMDS1 axis, distinct clustering patterns were evident in Northernmost Island and Wahlenbergfjorden, from Trinity Island, Wahlbergøya, and the others. The NMDS2 axis confirmed the relative separation of Trinity Island, Wahlbergøya, and Wahlenbergfjorden and of Ny Ålesund and Wahlenbergfjorden. These spatial separations in community structures might correlate with the site characteristics (Fig. 1D and E). Nonetheless, some overlap microbiota profiles representing Ny-Ålesund and sites TI/WJ/WB indicated a degree of compositional similarity across these sites (Fig. S4a). Further, some sites showed the community structure within-site variability, e.g., in Ny-Ålesund. As different microorganisms may harbor different functional potentials, the partial overlap and/or differentiation among the communities across sites may imply overlap or different microbial metabolic potentials, which were next identified.

In parallel with the OTU methodological approach, the ASV-based profiles also resulted in NMDS with statistical significance among sites (Fig. S5a: *P* < 0.001), with weak stress and *R*^2^. The ASV-based compositions at the phylum level were consistent broadly with the OTU-based approach, such as Proteobacteria dominating across all sites, ranging from 44.16% ± 1.04% in Northernmost Island to 72.40% ± 3.96% in Wahlenbergfjorden (Fig. S5b and Table S7). Actinobacteriota and Bacteroidota were the next abundant phyla. Site-specific microbial phylum patterns were also observed, such as those for Acidobacteriota that were enriched in Northernmost Island (39.91% ± 2.89%). The similarity between the ASV and OTU approaches extended to the genus-level analyses, with dominant genera including *Ilumatobacter*, *Sulfitobacter*, *Loktanella*, Flavobacteriaceae_unclassified, and Acidobacteriaceae_unclassified (Fig. S5c and Table S7). *Sulfurovum* was also detected in both datasets (classified under Campylobacterota in the ASV-based profiles).

### Microbial functional potentials and indications of site-associated environmental bacterial species signatures in the High Arctic soils

**Fig. 3.**
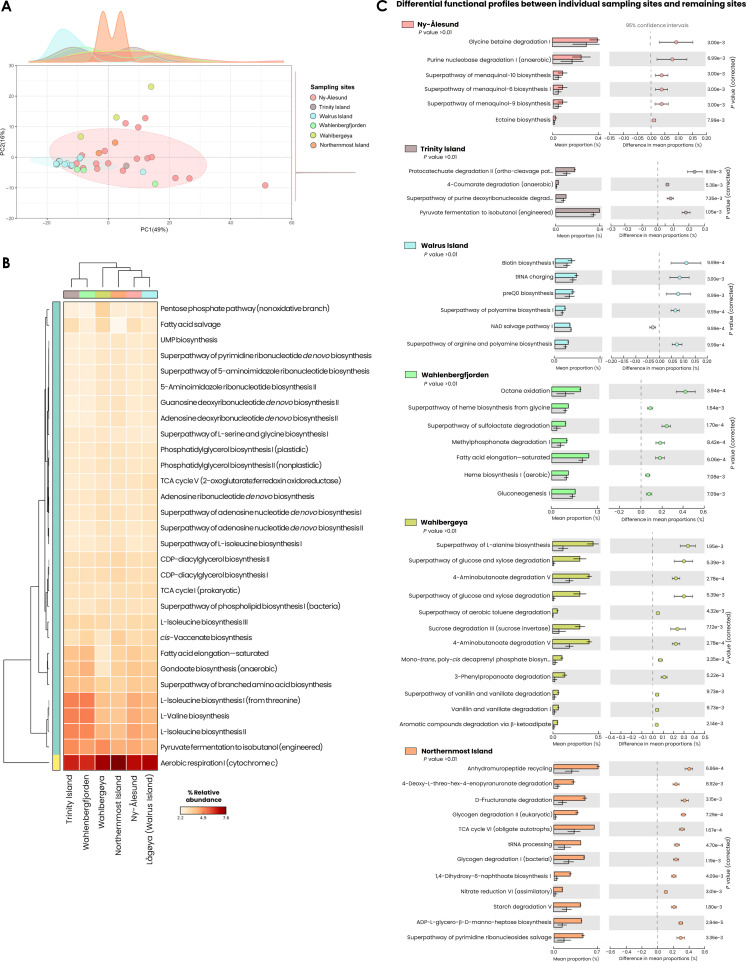
Estimated microbial function profiles based on MetaCyc pathways. (A) Principal component analysis (PCA), with density plots indicating the distributions across the PC1 and PC2 axes. (B) Heatmap representing relative percentages for different top 30 functions for each High Arctic microbiota soil sites, along (bottom horizontal axis) the clustering dendrogram of MetaCyc functions relatedness and (left vertical axis) the clustering dendrogram of microbial function potential profiles. Both dendrograms were constructed using the unweighted pair group method with arithmetic mean (UPGMA). (C) Statistically site-specific enriched function potential markers (MetaCyc pathways) (White’s nonparametric *t* test with Benjamini–Hochberg false discovery rate [FDR] correction, *P* < 0.01), between the selected site (color bar) and the average of the other sites (gray bar).

The functional profiles in the MetaCyc pathways of each soil site microbiota were estimated, and the principal component analysis of the predicted microbiota pathways revealed clustering patterns associated with sampling sites (Fig. 3A). The predominant microbial function potentials of the Svalbard soil microbiota were nucleotide and amino acid biosynthesis, lipid and membrane biosynthesis, and energy metabolism (Fig. 3B). Despite this common core, each site exhibited various enrichments of pathways (Fig. 3B): for instance, site Ny-Ålesund exhibited unique enrichment of glycine betaine degradation, purine nucleobase degradation, superpathways of menaquinol-6, menaquinol-9 and menaquinol-10 biosynthesis, and ectoine biosynthesis; Trinity Island exhibited protocatechuate degradation, 4-coumarate degradation, and pyruvate fermentation to isobutanol, consistent with complex organic compound turnover; Wahlbergøya exhibited the superpathway of l-alanine biosynthesis, the superpathway of glucose and xylose degradation, 4-aminobutanoate degradation, and several other pathways of aromatic compound and vanillin/vanillate degradations; and Northernmost Island was enriched in anhydromuropeptide recycling, enopyranuronate degradation, many glycogen and starch degradations, transfer RNA (tRNA) processing, and nitrate reduction, than the others (Fig. 3C), consistent with the clustering dendrogram of microbial function potential profiles among sites in Fig. 3A. Table S8a further demonstrates the relative percentages of the estimated functions of the top 30 abundances, confirming that the microbial functions (or metabolisms) involved in environmental processing were critical. The NSTI values, where a lower NSTI indicates higher confidence, were 0.43 ± 0.16 on average (Table S8b). Note that many novel (or unclassified) taxa (Fig. S3) could affect the low-confidence NSTI scores (e.g., WI2 and NA4).

**Fig. 4.**
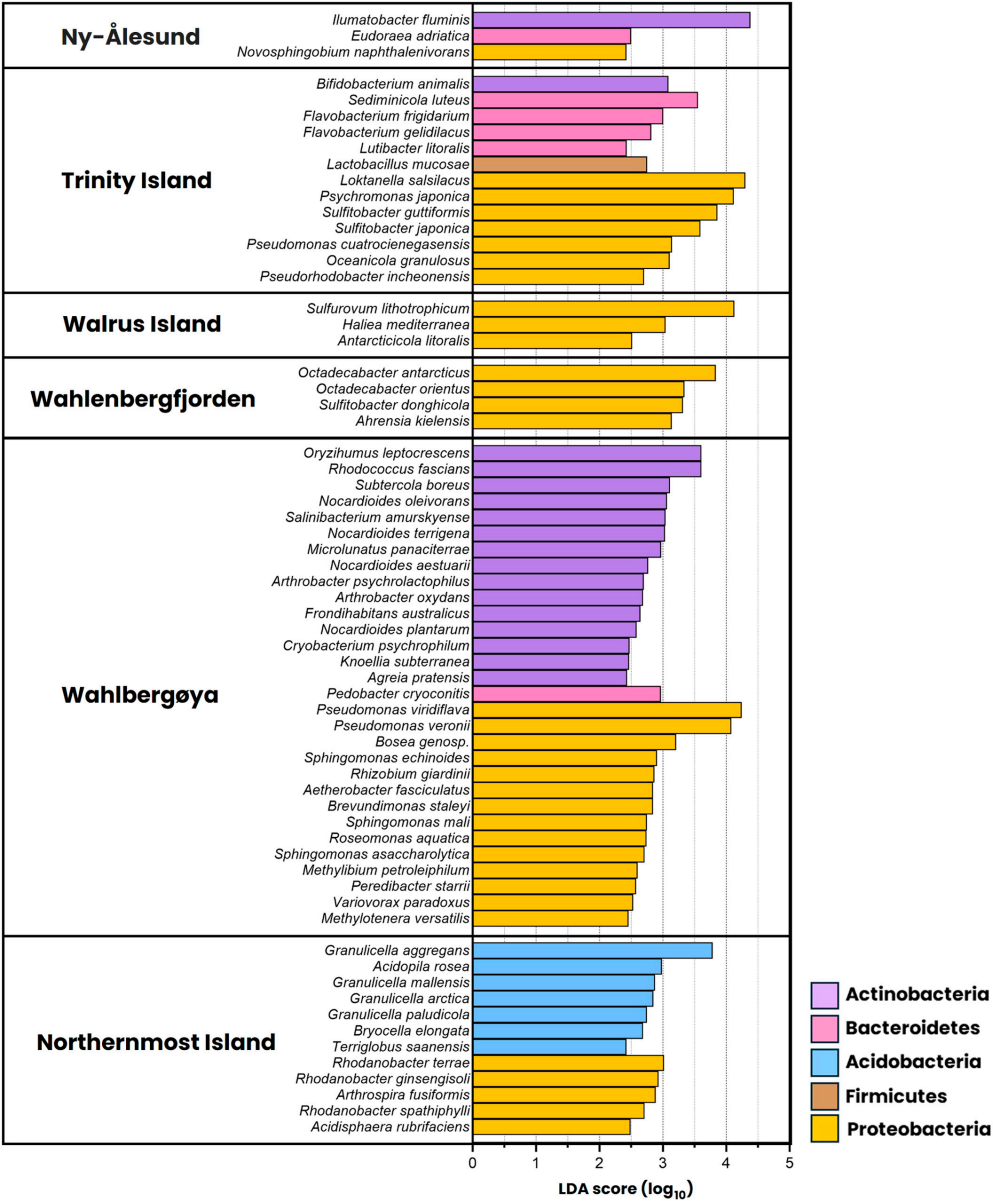
Linear discriminant analysis effect size (LEfSe) identifying site-specific differentially abundant bacterial species. All of Mothur 1.47.0’s standard operating procedure (SOP) classifiable species were included in this analysis; bar length defines the effect size of each species (only linear discriminant analysis [LDA] scores >2.4 are displayed), and bar color corresponds to phylum (Proteobacteria, Actinobacteria, Bacteroidetes, Acidobacteria, or Firmicutes).

Using LEfSe to determine candidate site-specific bacterial species taxa suggestive of site-specific environmental influences disclosed taxa potentially driven via environmental selection. Site Ny-Ålesund was characterized by taxa best matching *Novosphingobium naphthalenivorans*, *Eudoraea adriatica*, and *Ilumatobacter fluminis*, which are members of lineages previously associated with hydrocarbon degradation, marine organic matter turnover, and cold-environment adaptation, respectively; Trinity Island was characterized by coastal/psychrophilic lineages including *Loktanella salsilacus*, *Sulfitobacter* sp., *Psychromonas japonica*, and several *Flavobacterium* spp., with 2 taxa best matching *Bifidobacterium animalis* and *Lactobacillus mucosae* were also detected, potentially reflecting transient anthropogenic influence, although this interpretation remains speculative; and Walrus Island contained taxa related to sulfur-oxidizing marine bacterial lineages (*Sulfurovum lithotrophicum* and *Haliea mediterranea*) and *Antarcticicola litoralis* (Fig. 4) [89–101]. Wahlenbergfjorden also featured typical cold marine and sulfur-oxidizing bacteria (i.e., *Octadecabacter antarcticus* and *Sulfitobacter* sp.) [95,96,102,103]. In contrast, site Wahlbergøya showed a terrestrial signature with pollutant- and plant-associated degraders such as *Methylibium petroleiphilum*, *Pseudomonas veronii*, and *Variovorax paradoxus* alongside diverse Actinobacteria (*Rhodococcus* and *Nocardioides*) [104–106]. Lastly, site Northernmost Island was distinguished by Acidobacteria (*Granulicella*, *Terriglobus*, and *Acidipila*) and acid-tolerant Alphaproteobacteria *Acidisphaera* (Fig. 4 and Fig. S4b), consistent with oligotrophic, acidic soils [107].

### Conserved core microbiota compositions and their species co-occurrence patterns across the High Arctic sites

**Fig. 5.**
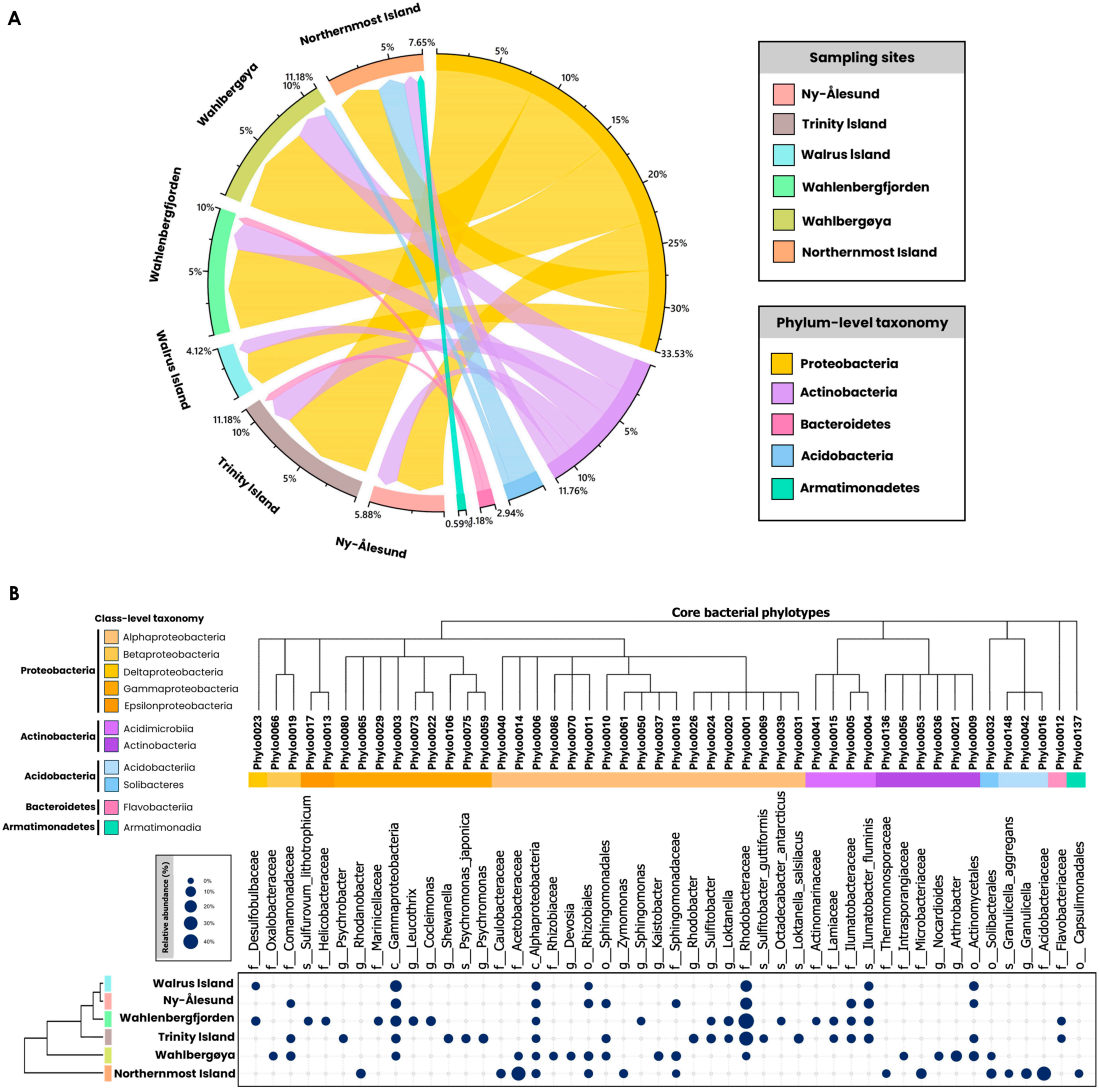
Site–taxon structure and core bacterial phylotypes across High Arctic sites of the Svalbard. (A) Chord diagram linking sampling sites to phylum-level core microbiota. Ribbon width is proportional to the summed relative abundance of core phylotypes from each phylum. Outer tick marks indicate the cumulative relative abundance to each sector. Site and phylum colors follow the keys at right. (B) Bubble plot demonstrating the relative percent abundances of each core phylotype (columns) for each site (rows), with the left dendrogram clustering sites via similarity in their core microbiota profiles and the top dendrogram clustering phylotypes via hierarchical classification from phylum to species (the colored bar infers class-level taxonomy). Bubble size indicates percentage abundance. Note that Mothur 1.47.0’s identification of OTUs as taxa f__koll13, f__JdFBGBact, f__C111, and o__FW68 utilized BLASTN *vs*. the NCBI nonredundant database, and the taxa are reported in this figure as f__Actinomarinaceae, f__Lamiaceae, f__Ilumatobacteraceae, and o__Capsulimonadales, respectively, to replace uncommon names.

Analysis of core microbiota compositions showed the remaining predominance by Proteobacteria, which contributed the largest proportion of core phylotypes for every High Arctic site. Actinobacteria contributed smaller but consistent fractions, whereas Bacteroidetes, Acidobacteria, and Armatimonadetes were minor and site restricted (Fig. 5A). The relative percent dendrogram of each phylotype showed predominance of Proteobacteria, Actinobacteria, and Acidobacteria in the High Arctic soils, allowing clear separation of phylotype patterns for Northernmost Island from Wahlbergøya and then Trinity Island from Wahlenbergfjorden, and Ny-Ålesund and Walrus Island were relatively the closest (Fig. 5B: left dendrogram). The findings were consistent with the AMOVA and PERMANOVA statistics of the core microbiota, reporting *P* < 0.001 among 6 sites (Table S6).

**Fig. 6.**
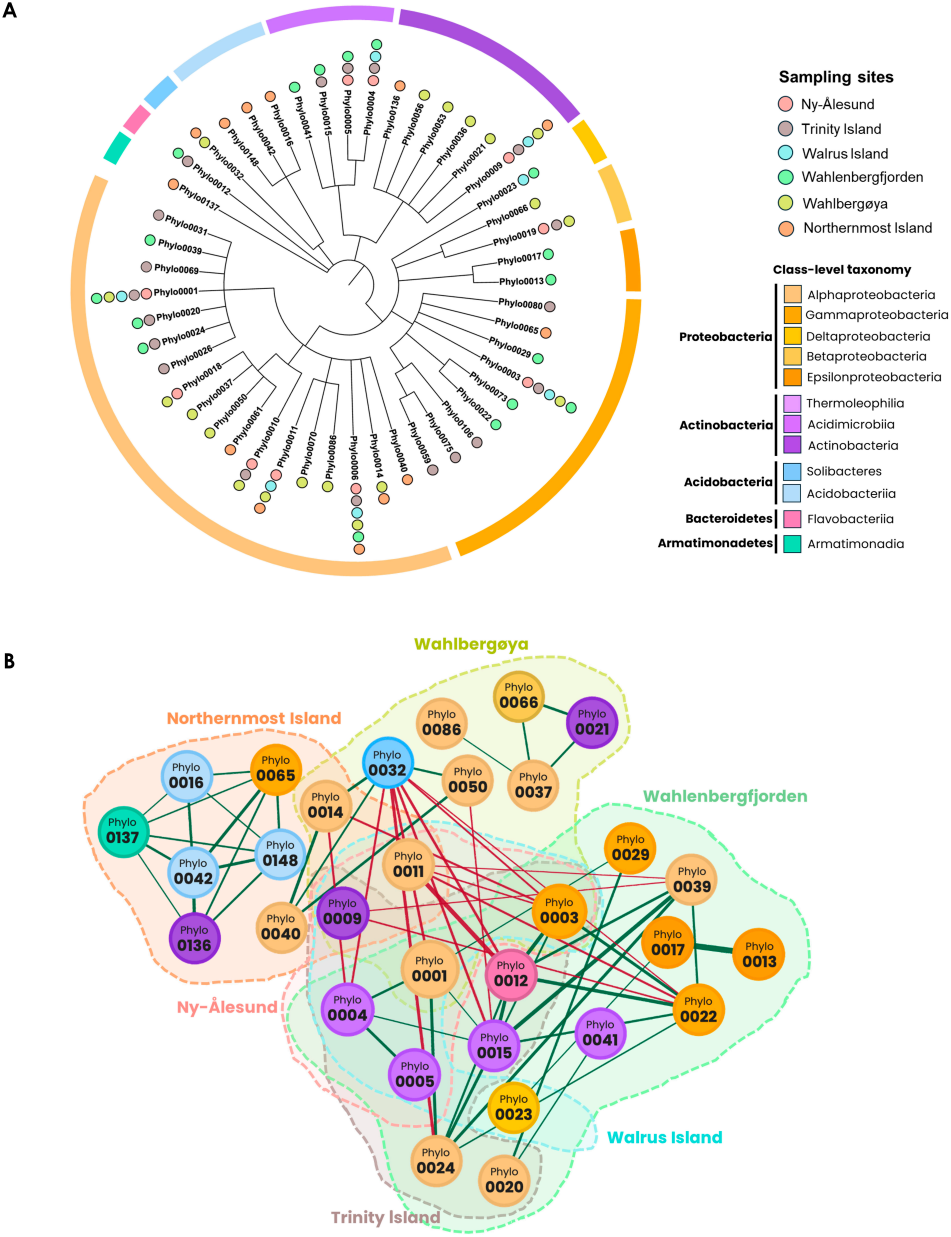
Taxonomic relationships and site-specific co-occurrence of core bacterial phylotypes in the High Arctic sites of the Svalbard. (A) Circular phylogenetic dendrogram of 48 core phylotypes, displaying hierarchical classification from phylum to species. Colored dots adjacent to each tip denote the sampling sites (Ny-Ålesund, Trinity Island, Walrus Island, Wahlenbergfjorden, Wahlbergøya, and Northernmost Island) in which the phylotype was detected as core. Outer ring segments indicate phylum-level groupings, with colors corresponding to Proteobacteria, Actinobacteria, Acidobacteria, Bacteroidetes, and Armatimonadetes. (B) Sparse Correlations for Compositional data (SparCC)-derived co-occurrence network of the same core species phylotypes (|*ρ*| > 0.8, false discovery rate [FDR]-adjusted *P* < 0.001) among sites; only phylotypes and correlations meeting these thresholds are displayed. Nodes are colored by taxonomic classes. The statistically significant associations are shown: positive correlations as green connecting lines, and negative correlations as red connecting lines; line width reflects the correlation magnitude; and colored dashed polygons group phylotypes according to their site-specific core distributions. In (A) and (B), species phylotypes that could not be classified by species names are denoted by the lowest identifiable taxon (o_ for order, f_ for family, g_ for genus, and s_ for species).

The circular dendrogram indicated a 48-species core spanning 5 phyla: Proteobacteria dominated (32 out of 48 species: 66.77%), followed by Actinobacteria (10 of 48: 20.83%), Acidobacteria (4 of 48: 8.33%), and single representatives of Bacteroidetes and Armatimonadetes (each 1 of 48: 2.1%) (Fig. 6A and Table S8a). Within Proteobacteria, Alphaproteobacteria were the most prevalent, with additional contributions from Gamma-, Beta-, Epsilon-, and Deltaproteobacteria. One unclassified Alphaproteobacteria phylotype (Phylo0006) (BLASTN *E* value 0.00 as *Parasphingorhabdus*) was consistently detected across all sampled sites and may represent a conserved core lineage within this Svalbard soil dataset. Actinobacteria were represented by 12 phylotypes belonging to the classes Acidimicrobiia and Actinobacteria. Acidobacteria were represented by 1 Solibacteres and 3 Acidobacteriia lineages and are restricted only to sites Northernmost Island and Wahlbergøya. Bacteroidetes and Armatimonadetes species were present only in Wahlenbergfjorden, Trinity Island, and Northernmost Island sites (Fig. 6A).

To ensure the reliability of the taxonomic trends for the genus (and species) levels, the microbial communities were evaluated at the family level and compared for consistency. The core phylotype analysis at the family level (Fig. S6) revealed a distribution pattern consistent with the observations at the species level (Fig. 5). Briefly, Proteobacteria predominated as a core phylotype across all sites, mostly represented by the families *Sphingomonadaceae* and *Rhodanobacteraceae*, Rhizobiales, and Alphaproteobacteria; meanwhile, Actinobacteria (e.g., *Iumatobacteraceae*) were core but contributed smaller and consistent fractions across the sites. In contrast, *Flavobacteriaceae* (Bacteroidetes), *Solibacteraceae* (Acidobacteria), and *Capsulimonadaceae* (Armatimonadetes) exhibited site-restricted distributions (Fig. S6). Hence, in agreement with the species-core phylotype analysis, Northernmost Island was clearly separated from Wahlbergøya, followed by the separation of Trinity Island from Wahlenbergfjorden, while Ny-Ålesund and Walrus Island were relatively close in their core phylotype profiles (Fig. S6b).

The SparCC species-phylotype co-occurrence network resulted in 31 nodes and 62 connections (comprising 46 positive and 16 negative links) after establishment of filtering (|*ρ*| > 0.8; FDR-adjusted *P* < 0.001) (Fig. 6B and Table S9) [67,74]. The network had an average degree of 4.00 and a modularity index of 0.55, indicating structured communities with distinct functional modules [108,109]. Positive associations comprised 74.19% (46 of 62 connections), and negative associations comprised 25.81% (16 of 62). Proteobacterial and actinobacterial taxa appeared as major network hubs and contributed substantially to overall networks. Network modularity qualitatively aligned with sampling sites: Wahlenbergfjorden and Wahlbergøya displayed the densest, most overlapping clusters; Ny-Ålesund, Trinity Island, and Walrus Island showed intermediate connectivity; and Northernmost Island formed the sparsest, most weakly connected subnetwork. At Ny-Ålesund, the core network was largely confined to interactions among Proteobacteria (mainly Alpha- and Gammaproteobacteria) and Actinobacteria. Trinity Island showed a denser network with mixed positive and negative correlations centered on Alphaproteobacteria and Actinobacteria. The maximum highly connected networks occurred at Wahlenbergfjorden and Wahlbergøya. Northernmost Island was dominated by strong associations among Acidobacteria and Actinobacteria; within Acidobacteria, class Acidobacteriia occurred as a site-unique core (whereas Solibacteres also appeared at Wahlbergøya), and a rare Armatimonadetes lineage (o_FW68, BLASTN *E* value 0.00 as *Capsulimonas*) was present (Fig. 6 and Table S9).

### Linking core microbiota to potential functions: 4-part strategies of the Svalbard soil resilience

**Fig. 7.**
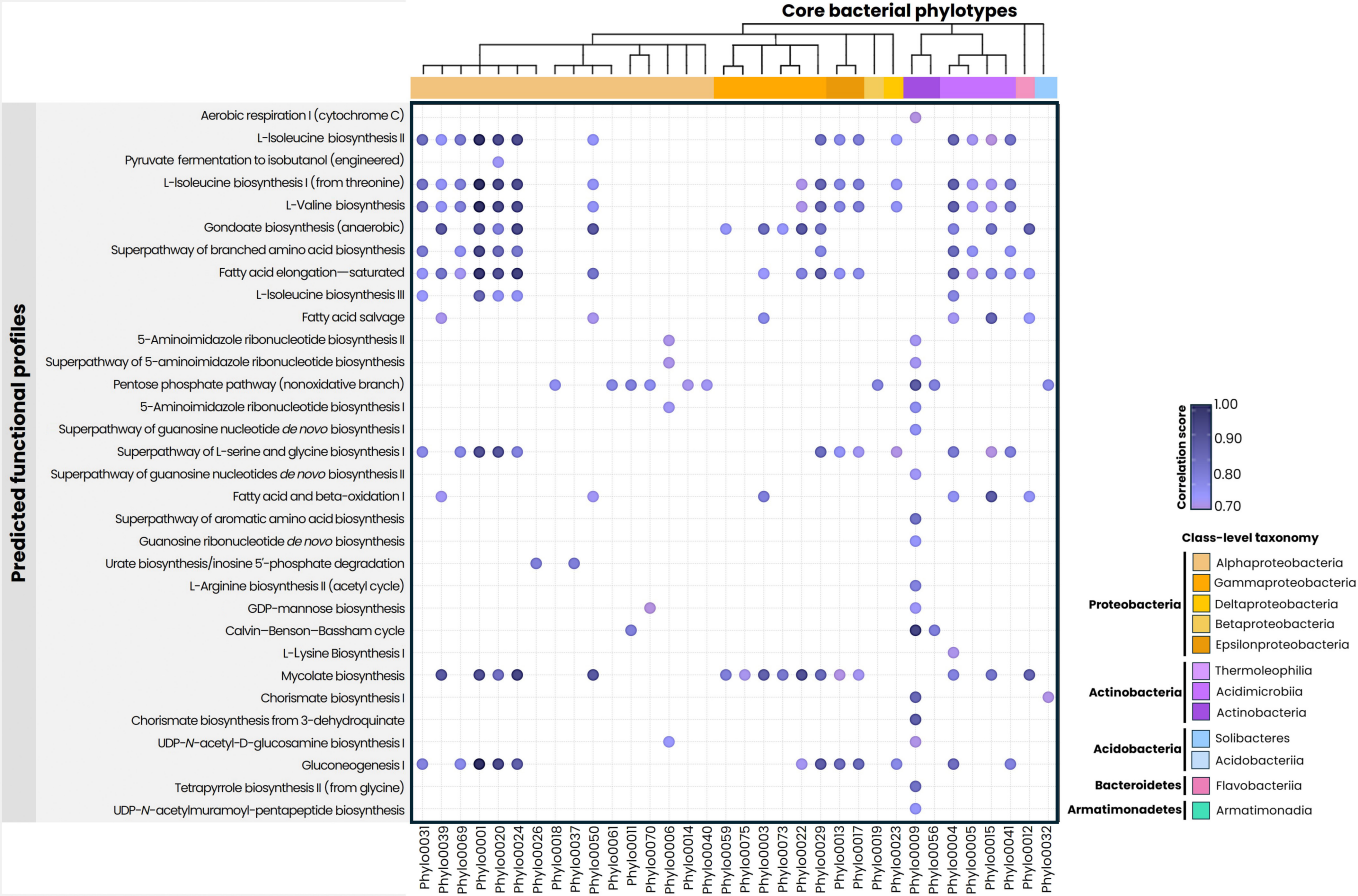
Spearman correlation of core bacterial phylotypes and predicted MetaCyc pathways in the High Arctic soils (*ρ* > 0.8, false discovery rate [FDR]-adjusted *P* < 0.001). Columns list phylotypes (color coded by phylum: Proteobacteria, Actinobacteria, Bacteroidetes, and Acidobacteria); rows list MetaCyc pathways. Dot color reflects the correlation coefficient (*ρ*), and only significant associations are displayed.

Linking core microbiota to potentially related MetaCyc pathways (Fig. 7) revealed 4 predicted functional themes potentially associated with persistence of the Svalbard soil microbiota under High Arctic conditions: (a) genome maintenance and nucleotide biosynthesis functions were widely associated with conserved core phylotypes distributed across all sites (Figs. 6 and 7). Examples included 5-aminoimidazole ribonucleotide (AIR) biosynthesis I and II, superpathway of AIR biosynthesis, guanosine nucleotide *de novo* biosynthesis I and II, and the nonoxidative branch of the pentose phosphate pathway. Broadly distributed Alphaproteobacteria core phylotypes, particularly unclassified Alphaproteobacteria Phylo0006 (Table S9a and Fig. 6A), showed strong positive associations with these pathways. These functions are involved in nucleotide precursor generation and cellular redox metabolism, suggesting conserved capacities related to DNA synthesis, repair, and genome stability under recurrent freeze–thaw and low-temperature conditions [110,111]. (b) Membrane remodeling and cell-envelope adaptation pathways were broadly linked to the predicted pathways, including fatty acid elongation, fatty acid salvage, fatty acid β-oxidation, gondoate biosynthesis, mycolate biosynthesis, and UDP-*N*-acetylmuramoyl-pentapeptide biosynthesis [112–115]. These pathways were associated with widespread taxa such as the dominant Proteobacteria and Actinobacteria core taxa (Fig. 6B) and also the unclassified *Rhodobacteraceae* (Phylo0001) and *I. fluminis* (Phylo0004), particularly across Ny-Ålesund, Trinity Island, Walrus Island, Wahlbergøya, and Wahlenbergfjorden (Fig. 6A). In addition, l-isoleucine and l-valine biosynthesis pathways linked to *Rhodobacteraceae*-associated phylotypes (Phylo0031, Phylo0039, Phylo0069, and Phylo0001) may contribute to branched-chain fatty acid production, whereas fatty acid salvage and β-oxidation pathways associated with Phylo0039, Phylo0050, Phylo0003, and Phylo0004 suggested membrane lipid remodeling capacities [116,117]. (c) Oxidative stress alleviation pathways, including predicted tetrapyrrole biosynthesis, pentose phosphate metabolism, GDP-mannose biosynthesis, and amino sugar and nucleotide-sugar metabolisms [118–121]. These functions were associated with widespread Alphaproteobacteria phylotypes (Phylo0018, Phylo0061, Phylo0011, Phylo0070, Phylo0014, and Phylo0040) and Actinobacteria Phylo0009 (Fig. 6). The linked pathways are generally associated with redox balance, antioxidant-related metabolism, and synthesis of cellular structural components, and their broad distribution across interconnected core taxa suggested another conserved resilience strategy shared among High Arctic microbiota. (d) Nutrient acquisition and degradation of alternative or recalcitrant substrates were associated with several core Alphaproteobacteria and Acidimicrobiia phylotypes. Predicted pathways included fatty acid salvage, fatty acid β-oxidation, glucose and xylose degradation, and degradation of aromatic compounds [122,123]. These functions were linked to Alphaproteobacteria Phylo0039 and Phylo0050 and Acidimicrobiia Phylo0005 and Phylo0041 (Fig. 6A). These predicted functions suggest metabolic flexibility for utilization of heterogeneous carbon sources in nutrient-limited Arctic soils, with additional pathways for the utilization of specific recalcitrant substrates associated with localized sites.

Together, integration of the core microbiota composition (core phylotypes), co-occurrence network structure (Fig. 6), and predicted functional associations (Fig. 7) revealed 4 complementary functional themes consisting of genome maintenance, membrane adaptation, oxidative-stress-associated metabolism, and flexible substrate utilization. These functions were distributed among conserved and interconnected core phylotypes across the Svalbard sites, suggesting shared potentially functional capacities associated with microbial persistence under High Arctic environmental and seasonal conditions.

## Discussion

This study demonstrated that High Arctic soils across the Svalbard harbor a conserved bacterial core superimposed on geographically structured, site-specific assemblages. The predominance of Proteobacteria across all sites corroborated their well-documented ecological versatility in polar ecosystems, with metabolic functions supporting persistence under chronic cold, oligotrophy, and freeze–thaw dynamics [17,29,124,125]. Actinobacteria and Acidobacteria displayed stronger spatial heterogeneity, consistent with environmental filtering along local edaphic gradients. The enrichment of Actinobacteria at Wahlbergøya supports their role in the decomposition of structurally recalcitrant substrates such as cellulose and chitin [126–128], whereas Northernmost Island exhibited relatively low alpha-diversity across all indices, and the dominance of Acidobacteria at Northernmost Island might reflect adaptation to acidic, nutrient-depleted soils typical of extreme Arctic terrestrial niches [129–131]. Meanwhile, Bacteroidetes, although less abundant, were relatively enriched at Ny-Ålesund and Walrus Island. Their known ability to degrade high-molecular-weight polysaccharides and adapt to moist or anaerobic niches likely facilitated their activity during thaw periods when labile carbon becomes available [125,132].

At finer taxonomic resolution, LEfSe analysis identified hypothesis-generating ecological signatures that likely reflected marine influence, nutrient regime, and potential anthropogenic inputs. LEfSe results were interpreted as exploratory ecological indicators rather than definitive biomarkers because they integrate statistical significance with effect-size estimation, facilitating identification of taxa that most strongly discriminate among soil communities; subsequently, as differential abundance results in microbiota datasets may vary depending on the normalization strategy, LEfSe may identify a larger number of significant taxa compared with more conservative compositional methods such as analysis of compositions of microbiome with bias correction (ANCOM-BC) [133,134]. Thus, the LEfSe candidate indicators should be considered together with beta-diversity, taxonomic profiles, and ecological functional patterns. Here, coastal sites were characterized by *Rhodobacteraceae*- and sulfur-associated lineages, consistent with potential influences from marine-derived organic matter and sulfur-related metabolisms [103,135]. In contrast, Wahlbergøya exhibited enrichment of metabolically versatile copiotrophs, including taxa previously reported from hydrocarbon-associated environments, suggesting enhanced processing of aromatic or plant-derived compounds [123,136]. Northernmost Island was distinguished by taxa commonly associated with acidic and oligotrophic environments, i.e., unclassified *Acetobacteraceae*, *Ilumatobacter*, and Acidobacteriaceae. These patterns were consistent with potential environmental filtering under low-nutrient and possibly acidic or saline conditions; however, the absence of direct soil physicochemical measurements, such as pH, moisture, salinity, and nutrient concentrations, limited ecological interpretation of the drivers underlying community assembly and functional differentiation across sites [137,138]. Collectively, these patterns supported the view that the High Arctic soil microbiota was not taxonomically unique relative to global soils [124] and was structured by intensified environmental filters and functional specialization at local scales. In addition, the co-occurrence network analysis should be interpreted cautiously because correlation-based associations inferred from SparCC do not necessarily represent direct ecological interaction or metabolic dependence observations, but shared environmental preferences or parallel responses in unmeasured environmental conditions.

Functional prediction analyses revealed hierarchical and local ecological organizations consisting of conserved metabolic functions and superimposed local enrichments. Potential core functions, including lipid and membrane biosynthesis, energy metabolism, and xenobiotic or aromatic degradation, likely represented universal requirements for persistence under prolonged freezing and episodic thaw [19]. Superimposition of the core functions with site-specific enrichments of xenobiotic degradation, redox metabolism, and/or phosphotransferase systems reflected local dynamic resource availability and environmental stressors. For example, Ny-Ålesund was characterized by osmoprotection and metabolic maintenance, ectoine biosynthesis, and glycine betaine degradation, supporting a strategy for stabilizing cellular components against the osmotic fluctuations common in Arctic soils [139,140]. For Trinity Island, a high capacity was predicted for the breakdown of complex aromatic substrates: e.g., the protocatechuate and 4-coumarate degradation pathways suggested involvement in the degradation of lignin-derived compounds and other recalcitrant matters. The pyruvate fermentation pathway suggested metabolic versatility for specialized carbon cycling [141,142]. Walrus Island highlighted a cellular maintenance and protein synthesis efficiency via biotin and preQ0 biosynthesis and active tRNA charging, in addition to arginine and polyamine biosynthesis that may assist a cold-adaptation strategy as polyamines are crucial for stabilizing macromolecular structures and mitigating freezing-induced stress [143,144]. For Wahlenbergfjorden, octane oxidation and sulfolactate degradation were predicted, providing utilizing capability of diverse organic and hydrocarbon substrates, complemented with an active heme biosynthesis pathway, which may reflect enhanced respiratory potential. The predicted pathways of fatty acid elongation and gluconeogenesis also expressed strategies for membrane stabilization and carbon homeostasis, potentially contributing to microbial persistence in the nutrient-limited and cold-stressed environment of the High Arctic [122,123,145–148]. Wahlbergøya demonstrated potential extended capacities of carbohydrate scavenging and aromatic degradation such as glucose, xylose, and sucrose, alongside vanillin and 3-phenylpropanoate degradation and l-alanine biosynthesis, underlying the predicted metabolic flexibility of the microbiota community to utilize diverse carbon sources and maintain cellular integrity in nutrient-limited Arctic soils [122,143]. Northernmost Island showed a distinct functional focus on resource conservation and energy recycling through enrichment of anhydromuropeptide recycling and glycogen/starch degradation pathways, nitrate reduction, and tRNA processing, which all suggested functions potentially associated with nutrient assimilation and translational maintenance in extreme environments like the terrestrial Arctic [144,149].

Integration of the core microbiota analysis with predicted MetaCyc pathways suggested 4 complementary functional resilience themes that might contribute to microbial persistence in High Arctic soils. First, genome integrity maintenance (via pathways associated with DNA replication, recombination, and repair; AIR biosynthesis; guanosine nucleotide *de novo* biosynthesis; and pentose phosphate pathway) may support cellular recovery from freeze–thaw-induced DNA damage and desiccation stress [110,111]. Second, membrane remodeling and cell-envelope adaptation functions, including fatty acid elongation, fatty acid salvage, β-oxidation, gondoate biosynthesis, mycolate biosynthesis, branched-chain amino acid biosynthesis, and UDP-*N*-acetylmuramoyl-pentapeptide biosynthesis, suggested dynamic restructuring of membrane lipids and envelope components potentially involved in maintaining membrane fluidity and cellular integrity under subzero and fluctuating Arctic conditions [112–117,150]. Third, oxidative-stress-associated pathways, including amino sugar and nucleotide-sugar biosynthesis, GDP-mannose metabolism, UDP-linked amino sugar, and biofilm-associated functions, may contribute to cellular protection and extracellular polysaccharide production that potentially enhance water retention, nutrient capture, and physical protection during thaw-associated reactive oxygen species pulses [120,121]. Fourth, resource acquisition and utilization of recalcitrant substrates, including phosphotransferase systems, d-amino acid metabolism, and pathways involved in the degradation of glucose, xylose, aromatic compounds, and xenobiotics, suggested metabolic flexibility for nutrient scavenging under chronic carbon limitation [122,123]. Importantly, these predicted functions were distributed among broadly shared core taxa, indicating that resilience-associated traits were not restricted to a few specialized lineages but instead embedded within the conserved microbial community structure across Svalbard soils. Nonetheless, these interpretations remain predictive and hypothesis generating because the functional profiles were inferred from 16S rRNA gene-based analyses rather than directly validated through metagenomic, transcriptomic, or physiological approaches. In addition, the lack of environmental metadata, including physicochemical measurements such as pH, moisture, salinity, nutrient availability, and organic carbon content, limited direct evaluation of the environmental drivers shaping microbial community composition and predicted functional organization across Svalbard soils. Demonstrating microbial resilience in Arctic soils would therefore require integrated longitudinal sampling, environmental characterization, and direct functional validation through metagenomic, transcriptomic, metabolomic, or ecosystem process measurements. Therefore, the functional patterns identified here should be interpreted as putative ecological strategies consistent with microbial persistence under High Arctic environmental conditions rather than direct evidence of adaptive resilience mechanisms.

Co-occurrence network topology further supported the presence of a conserved bacterial framework across sites, with Proteobacteria and Actinobacteria acting as major network hubs, forming predominantly positive associations in consistent with cooperative or co-selected ecological interactions [151]. Negative correlations, for instance, between copiotrophs and oligotrophs such as Acidobacteria, suggested niche segregation driven by specific resource or physiological conditions [151–153]. Sites with higher connectivity (Wahlbergøya and Wahlenbergfjorden) might infer greater metabolic complementarity or resource variability, whereas the sparsely connected Northernmost Island network suggested stronger abiotic constraint and niche segregation [154,155]. The persistence of a pan-core Alphaproteobacteria phylotype across all samples underscored the presence of broadly adapted taxa capable of occupying heterogeneous Arctic niches [124]. Yet, these interpretations remained tentative as unequal sample sizes and abundance filtering thresholds may partly influence network structure analyses.

Despite co-analyses with environmental parameters from satellite-derived habitat classification and ERA5-Land surface temperature profiles, a limitation of the study is the absence of direct soil physicochemical measurements, including moisture content, salinity, organic carbon, and nutrient concentrations. These environmental variables are capable as drivers of soil bacterial community composition and functional potential. Subsequently, interpretations regarding environmental filtering and local ecological selection in the present study remained inferential and based primarily on observed taxonomic, diversity, and predicted functional differences among sites, rather than on direct environmental–microbial association analyses. While the geographically structured microbiota patterns strongly suggested site-level environmental influences, definitive attribution of these patterns to specific abiotic drivers remains beyond the scope of the current dataset. Future studies integrating paired microbiota and soil physicochemical measurements will be essential to validate the mechanisms underlying environmental selection and microbial resilience across Svalbard soils.

Overall, the present study provided evidence for spatial structuring and conserved bacterial core across Svalbard soils. These findings supported a model in which geographically distinct communities retained shared taxonomic components and predicted functional potentials, while local differences may reflect site-specific selective pressures. Such a configuration might also confer ecosystem stability under rapid Arctic warming, yet there were localized functional shifts, particularly in carbon and xenobiotic metabolism [125,156–158]. Still, the functional predictions were mere inferences from 16S rRNA gene data and required supportive datasets of metagenomic or active RNA extraction followed metatranscriptomic studies, and the marine microbiota near the shoreline sites may in the future be analyzed to understand any correlation. Yet, convergence results across independent analytical frameworks strengthened the ecological inferences of the High Arctic soils microbiota as governed by core stability coupled with modular functional plasticities that may be related to resource availability and/or ecosystem responses under accelerating climate change [19,29,157,159,160]. The results provided significant knowledge useful toward sustainable polar-environment monitoring and management.

## Conclusions

Soil bacterial communities across 6 High Arctic Svalbard sites exhibited spatial structuring together with a conserved taxonomic and functional core overlaid by geographically structured specialization. The presence of a 48-phylotype shared core and site-specific putative biomarker taxa indicates that both common and geographically distinct community components contribute to microbiota organization. Concurrently, site-specific biomarkers and functional enrichments revealed environmental filtering for localized adaptations. Predicted functional analyses further suggested conserved metabolic functions related to maintenance and stress-associated pathways, alongside site-specific enrichments in substrate utilization and redox-related processes. However, because these functional profiles were inferred from 16S rRNA gene sequencing, this core–periphery configuration was interpreted as putative functional potentials rather than direct evidence of resilience, buffering capacity, or realized ecological function. The convergence findings across taxonomic profiles, beta-diversity, hypothesis of microbiota core, co-occurrence network, and inferred functional analyses strengthened the ecological interpretation; meanwhile, functional predictions require validation through shotgun metagenomics and transcriptomics. Overall, the findings provided testable hypotheses for appropriate stress-adapted capability while permitting spatially tuned functional reconfigurations during current Arctic climate change. Yet, the ecological interpretations presented here remain constrained by the absence of direct environmental measurements and experimentally validated functional data, highlighting the need for future studies integrating soil physicochemical characterization with multi-omics and ecosystem-level analyses.

## Data Availability

The 16S rRNA gene sequences in this study were deposited in the NCBI open access Sequence Read Archive database (accession number PRJNA1427094).
