# Supplementary material for: Core–Periphery Microbiota Organization Is Associated with Functional Resilience in High Arctic Svalbard Soils
Source: Comput Struct Biotechnol J. 2026 Jun 24;35(1):0135. doi: 10.34133/csbj.0135 (PMC13484907; doi:10.34133/csbj.0135)
Supplement: Supplementary file 1 [file csbj.0135.f1.zip › Supplementary Materials.docx]

**Supporting information**

**Fig. S1.** Rarefaction curves illustrating phylotype richness for each sample at the phylum and genus levels. The curves demonstrated sequencing depth and coverage across samples, indicating the adequacy of the sequences in capturing bacterial diversity.

**Fig. S2.** Violin plots showing comparative average alpha diversity indices including Chao1 richness, Shannon diversity, and inverse Simpson diversity of microbial communities across 6 soil locations, at genus level. Each color represents an exploratory sampling site, with individual points indicating diversity values within the site. The width of each violin reflects the density distribution of values. Diversity values at the genus level were from **Table S2**.

**Fig. S3. Average percent relative abundances of bacterial genera across all sample sites.** (**a)** Bar chart with genera that had < 1% were grouped under the category “Other genera (<1%)," genera that could not be confidently assigned phyla were under "Unclassified genera," and phylotypes were classified to the genus or the deepest level to which each phylotype could be reliably assigned to, with a lowercase letter represents taxonomic prefix (c_ for class; o_, order; f_, family, and g_, genus). **(b)** Heatmap of the top 20 abundant bacterial genera across sites. Genera were grouped by their respective phyla.

**Fig. S4.** (**a**) Non-metric multidimensional scaling (NMDS) at genus-level and (**b**) phylogenetic clustering at species-level, both constructed using Thetayc dissimilarity metrices.

**Fig. S5.** ASV-based bacterial community analysis across Svalbard sampling sites. (**a**) Non-metric multidimensional scaling (NMDS) at the genus level, and taxonomic compositions at (**b**) phylum and (**c**) genus levels.

**Fig. S6.** Site–taxon structure and core bacterial phylotypes at the family level across High Arctic sites of Svalbard. (**a**) Chord diagram illustrating the relationships between sampling sites and family-level core microbiota. Ribbon width is proportional to the summed relative abundance of core phylotypes assigned to each family. Outer tick marks indicate the cumulative relative abundance within each sector. Colors for sites and taxonomic groups correspond to the legends on the right. (**b**) Bubble plot showing the relative abundance (%) of each core family-level phylotype (columns) across sampling sites (rows). The left dendrogram represents hierarchical clustering of sites based on similarity in their core microbiota profiles, while the top dendrogram shows clustering of phylotypes according to taxonomic relationships. Bubble size is proportional to relative abundance.

**Table S1.** Geographic coordinates of the sampling sites, along the number of raw and quality sequencing reads.

| **Sample Id** | **Type of sample** | **Location site** | **Coordinates** | **Raw reads** | **Quality reads** |
| --- | --- | --- | --- | --- | --- |
| NA1_1 | Soil | Ny-Ålesund | 78°55'28.1"N, 11°53'28.2"E | 55967 | 51293 |
| NA1_2 | Soil | Ny-Ålesund | 78°55'28.1"N, 11°53'28.2"E | 73735 | 65349 |
| NA1_3 | Soil | Ny-Ålesund | 78°55'28.1"N, 11°53'28.2"E | 34505 | 31257 |
| NA2_1 | Soil | Ny-Ålesund | 78°55'28.1"N, 11°53'28.2"E | 74781 | 70040 |
| NA2_2 | Soil | Ny-Ålesund | 78°55'28.1"N, 11°53'28.2"E | 35388 | 33503 |
| NA2_3 | Soil | Ny-Ålesund | 78°55'28.1"N, 11°53'28.2"E | 132096 | 124583 |
| NA3_1 | Soil | Ny-Ålesund | 78°55'28.1"N, 11°53'28.2"E | 94704 | 86331 |
| NA3_2 | Soil | Ny-Ålesund | 78°55'28.1"N, 11°53'28.2"E | 103016 | 95605 |
| NA3_3 | Soil | Ny-Ålesund | 78°55'28.1"N, 11°53'28.2"E | 109334 | 99589 |
| NA4_1 | Soil | Ny-Ålesund | 78°55'28.1"N, 11°53'28.2"E | 57572 | 44239 |
| NA4_2 | Soil | Ny-Ålesund | 78°55'28.1"N, 11°53'28.2"E | 87959 | 71258 |
| NA4_3 | Soil | Ny-Ålesund | 78°55'28.1"N, 11°53'28.2"E | 21659 | 15422 |
| NA5_1 | Soil | Ny-Ålesund | 78°55'28.1"N, 11°53'28.2"E | 105653 | 100091 |
| NA5_2 | Soil | Ny-Ålesund | 78°55'28.1"N, 11°53'28.2"E | 191497 | 178651 |
| NA5_3 | Soil | Ny-Ålesund | 78°55'28.1"N, 11°53'28.2"E | 46083 | 43181 |
| NA6_1 | Soil | Ny-Ålesund | 78°55'28.1"N, 11°53'28.2"E | 80604 | 71872 |
| NA6_2 | Soil | Ny-Ålesund | 78°55'28.1"N, 11°53'28.2"E | 107084 | 88233 |
| NA6_3 | Soil | Ny-Ålesund | 78°55'28.1"N, 11°53'28.2"E | 101382 | 82632 |
| TI_1 | Soil | Trinity island | 79°33.496'N, 11°02.104'E | 93332 | 80343 |
| TI_2 | Soil | Trinity island | 79°33.496'N, 11°02.104'E | 28874 | 26592 |
| WI1_1 | Soil | Walrus island | 80°22.023'N, 18°19.124'E | 114989 | 107811 |
| WI1_2 | Soil | Walrus island | 80°22.023'N, 18°19.124'E | 34462 | 31941 |
| WI1_3 | Soil | Walrus island | 80°22.023'N, 18°19.124'E | 15581 | 13959 |
| WI2_1 | Soil | Walrus island | 80°22.023'N, 18°19.124'E | 48096 | 43901 |
| WI2_2 | Soil | Walrus island | 80°22.023'N, 18°19.124'E | 42378 | 39163 |
| WI2_3 | Soil | Walrus island | 80°22.023'N, 18°19.124'E | 16835 | 14606 |
| WI3_1 | Soil | Walrus island | 80°22.023'N, 18°19.124'E | 15979 | 12176 |
| WI3_2 | Soil | Walrus island | 80°22.023'N, 18°19.124'E | 25316 | 23389 |
| WI4_1 | Soil | Walrus island | 80°22.023'N, 18°19.124'E | 12961 | 11320 |
| WI4_2 | Soil | Walrus island | 80°22.023'N, 18°19.124'E | 20550 | 18785 |
| WJ_1 | Soil | Wahlenbergfjorden | 79°40.163'N, 19°43.772'E | 36515 | 33581 |
| WJ_2 | Soil | Wahlenbergfjorden | 79°40.163'N, 19°43.772'E | 29499 | 27441 |
| WJ_3 | Soil | Wahlenbergfjorden | 79°40.163'N, 19°43.772'E | 95013 | 90589 |
| WB_1 | Soil | Wahlbergøya | 79°21.01'N, 19°58.20'E | 38115 | 35962 |
| WB_2 | Soil | Wahlbergøya | 79°21.01'N, 19°58.20'E | 97268 | 92513 |
| WB_3 | Soil | Wahlbergøya | 79°21.01'N, 19°58.20'E | 72743 | 67897 |
| NI_1 | Soil | Northernmost Island | 80°49'24.2'N, 20°21'34.6'E | 53566 | 50681 |
| NI_2 | Soil | Northernmost Island | 80°49'24.2'N, 20°21'34.6'E | 66381 | 63897 |
| **Total** |  |  |  | 2471472 | 2239676 |
| **Average** |  |  |  | 65038.74 | 58938.84 |

**Table S2.** Summary of statistical analyses used in this research.

| **Analysis objective** | **Data type** | **Statistical method** | **Transformation / normalization** | **Multiple-testing correction** | **Assumptions** | **Software / package** |
| --- | --- | --- | --- | --- | --- | --- |
| Alpha diversity comparison | Chao1, Shannon, inverse Simpson indices | Kruskal–Wallis test followed by Dunn’s post-hoc test | Rarefied dataset (11,320 reads/sample) | Benjamini–Hochberg (BH) correction | Non-parametric approach used due to non-normal distribution | R v4.4.2 (FSA) |
| Beta diversity ordination | ThetaYC dissimilarity matrix | NMDS ordination | Rarefied dataset  (11,320 reads/sample) | Not applicable | Stress values used to evaluate ordination reliability | Mothur v1.47.0;  R v4.4.2 (ggplot2, ggExtra) |
| Taxonomic classification | OTU / ASV sequences | Naïve Bayesian classifier | SILVA v138.1 alignment | Not applicable | Bootstrap cutoff  = 80% | Mothur1.47.0 |
| Community structure comparison | ThetaYC dissimilarity matrix | AMOVA | Rarefied dataset  (11,320 reads/sample) | Permutation-based | 999 permutations | Mothur1.47.0 |
| Community structure comparison | ThetaYC dissimilarity matrix | PERMANOVA (adonis2) | Rarefied dataset (11,320 reads/sample) | Permutation-based | PERMDISP used to evaluate homogeneity of multivariate dispersion prior to PERMANOVA | R v4.4.2 (vegan) |
| Homogeneity of dispersion | ThetaYC dissimilarity matrix | PERMDISP (betadisper) | Rarefied dataset (11,320 reads/sample) | Permutation-based | Used to assess homogeneity of multivariate dispersion among groups | R v4.4.2 (vegan) |
| Differential abundance / biomarker detection | Relative abundance taxa | LEfSe | Relative abundance normalization from rarefied OTU table | Internal LEfSe  procedure | Exploratory biomarker discovery: LDA > 2.4,  *p* < 0.05 | Mothur1.47.0;  GraphPad Prism version 10.2.3 |
| Functional prediction | 16S OTU table  and representative sequences | PICRUSt2 | Rarefied OTU table | Not applicable | NSTI values reported to indicate prediction confidence | PICRUSt2 |
| Functional pathway comparison | PICRUSt2 MetaCyc  pathways | White’s non-parametric t-test | Relative abundance pathway profiles | Benjamini–Hochberg FDR | Appropriate for compositional microbiome proportion data | STAMP |
| Core taxa - predictive function association | Core phylotypes  vs MetaCyc pathways | Spearman correlation | Relative abundance data of OTU and predicted function table | FDR-adjusted *p*-values | ρ > 0.7, FDR-adjusted *p* < 0.05 | R v4.4.2 (Hmisc, stats, ggplot2, reshape2, dplyr) |
| Co-occurrence network construction | Core phylotypes | SparCC  correlation | Relative abundance data with filtering | FDR-adjusted *p*-values | \|ρ\| > 0.8, FDR–adjusted *p* < 0.001 | Mothur1.47.0;  R v4.4.2 (igraph) |
| Network analysis | Co-occurrence network | Louvain modularity algorithm | Filtered correlation network | Not applicable | Modularity index calculated from network topology | R v4.4.2 (igraph); Cytoscape v3.10.3 |

**Table S3.** Alpha diversity indices and Good’s coverage estimates for bacterial communities at (**a**) phylum and (**b**) genus levels across all samples, at normalized sequencing depth of 11,320 quality reads per sample.

(**a**)

| **Sample ID** | **Good’s coverage percentage** | **OTUs** | **Chao1** | **Shannon** | **Inv.Simpson** |
| --- | --- | --- | --- | --- | --- |
| NA1_1 | 99.97 | 17 | 17.75 | 1.22 | 2.71 |
| NA1_2 | 99.96 | 17 | 22.00 | 1.22 | 2.78 |
| NA1_3 | 99.99 | 14 | 14.00 | 1.22 | 2.73 |
| NA2_1 | 99.97 | 18 | 19.50 | 1.17 | 2.38 |
| NA2_2 | 99.95 | 19 | 24.00 | 0.67 | 1.45 |
| NA2_3 | 99.95 | 19 | 26.50 | 0.73 | 1.49 |
| NA3_1 | 99.96 | 18 | 28.00 | 1.16 | 2.35 |
| NA3_2 | 99.96 | 18 | 21.00 | 1.12 | 2.22 |
| NA3_3 | 99.99 | 16 | 16.00 | 1.16 | 2.36 |
| NA4_1 | 99.98 | 22 | 22.33 | 1.54 | 3.63 |
| NA4_2 | 99.99 | 21 | 21.00 | 1.55 | 3.56 |
| NA4_3 | 100.00 | 18 | 18.00 | 1.37 | 3.37 |
| NA5_1 | 99.97 | 17 | 20.00 | 0.86 | 1.80 |
| NA5_2 | 99.98 | 18 | 18.50 | 1.03 | 2.30 |
| NA5_3 | 99.94 | 20 | 41.00 | 1.00 | 2.28 |
| NA6_1 | 99.97 | 23 | 24.50 | 1.16 | 1.98 |
| NA6_2 | 99.96 | 24 | 26.00 | 1.27 | 2.21 |
| NA6_3 | 99.97 | 25 | 25.60 | 1.35 | 2.47 |
| TI_1 | 100.00 | 13 | 13.00 | 1.24 | 2.88 |
| TI_2 | 99.99 | 15 | 15.00 | 1.16 | 2.61 |
| WI1_1 | 99.99 | 18 | 18.00 | 1.16 | 2.38 |
| WI1_2 | 99.97 | 16 | 17.00 | 0.61 | 1.36 |
| WI1_3 | 99.97 | 18 | 19.00 | 0.78 | 1.58 |
| WI2_1 | 99.98 | 19 | 19.33 | 1.24 | 2.65 |
| WI2_2 | 99.96 | 21 | 23.00 | 1.27 | 2.69 |
| WI2_3 | 99.92 | 26 | 44.00 | 1.11 | 1.99 |
| WI3_1 | 99.94 | 26 | 33.00 | 1.08 | 1.86 |
| WI3_2 | 99.96 | 21 | 23.00 | 0.94 | 1.70 |
| WI4_1 | 99.97 | 24 | 25.00 | 1.85 | 5.00 |
| WI4_2 | 99.96 | 20 | 22.00 | 1.62 | 4.05 |
| WJ_1 | 99.96 | 19 | 22.00 | 1.45 | 3.55 |
| WJ_2 | 99.96 | 15 | 18.33 | 0.86 | 1.64 |
| WJ_3 | 99.99 | 16 | 16.00 | 0.82 | 1.63 |
| WB_1 | 100.00 | 10 | 10.00 | 1.11 | 2.27 |
| WB_2 | 99.97 | 12 | 13.50 | 0.92 | 1.79 |
| WB_3 | 99.96 | 18 | 19.50 | 0.90 | 1.70 |
| NI_1 | 99.97 | 18 | 18.75 | 0.74 | 1.47 |
| NI_2 | 99.96 | 18 | 21.00 | 0.73 | 1.46 |
| **Total** | 3798.91 | 707 | 816.10 | 42.38 | 90.31 |
| **Average** | 99.97 | 18.61 | 21.48 | 1.12 | 2.38 |

(**b**)

| **Sample ID** | **Good’s coverage percentage** | **OTUs** | **Chao1** | **Shannon** | **Inv.Simpson** |
| --- | --- | --- | --- | --- | --- |
| NA1_1 | 99.52 | 198 | 255.24 | 3.62 | 19.91 |
| NA1_2 | 99.62 | 183 | 233.17 | 3.64 | 20.94 |
| NA1_3 | 99.58 | 207 | 237.03 | 3.63 | 20.85 |
| NA2_1 | 99.28 | 281 | 362.00 | 3.62 | 16.19 |
| NA2_2 | 99.37 | 223 | 300.66 | 3.06 | 8.99 |
| NA2_3 | 99.31 | 242 | 327.80 | 3.20 | 10.66 |
| NA3_1 | 99.50 | 227 | 275.36 | 3.71 | 22.14 |
| NA3_2 | 99.42 | 235 | 296.29 | 3.68 | 20.65 |
| NA3_3 | 99.35 | 249 | 345.46 | 3.72 | 21.75 |
| NA4_1 | 99.26 | 277 | 397.21 | 3.76 | 17.10 |
| NA4_2 | 99.26 | 295 | 400.64 | 3.77 | 16.38 |
| NA4_3 | 99.45 | 235 | 289.03 | 3.12 | 8.10 |
| NA5_1 | 99.37 | 218 | 300.83 | 3.08 | 7.77 |
| NA5_2 | 99.27 | 253 | 362.77 | 3.27 | 11.36 |
| NA5_3 | 99.29 | 234 | 332.75 | 3.17 | 10.54 |
| NA6_1 | 98.93 | 412 | 529.10 | 4.29 | 32.68 |
| NA6_2 | 98.77 | 458 | 585.88 | 4.44 | 39.62 |
| NA6_3 | 98.81 | 461 | 588.39 | 4.47 | 40.65 |
| TI_1 | 99.11 | 307 | 408.00 | 3.70 | 16.99 |
| TI_2 | 99.36 | 223 | 298.18 | 3.17 | 9.64 |
| WI1_1 | 99.36 | 285 | 358.00 | 3.93 | 25.38 |
| WI1_2 | 99.32 | 289 | 377.67 | 3.85 | 21.34 |
| WI1_3 | 99.36 | 239 | 318.88 | 3.33 | 12.68 |
| WI2_1 | 99.29 | 326 | 424.75 | 3.99 | 21.44 |
| WI2_2 | 99.28 | 268 | 395.73 | 3.74 | 18.13 |
| WI2_3 | 99.39 | 222 | 300.20 | 2.96 | 6.94 |
| WI3_1 | 99.54 | 189 | 252.14 | 3.30 | 11.37 |
| WI3_2 | 99.60 | 192 | 231.60 | 3.60 | 16.57 |
| WI4_1 | 99.69 | 169 | 192.80 | 3.13 | 10.29 |
| WI4_2 | 99.55 | 177 | 232.43 | 3.14 | 10.41 |
| WJ_1 | 99.39 | 203 | 289.89 | 3.19 | 9.07 |
| WJ_2 | 99.40 | 197 | 310.90 | 3.01 | 7.11 |
| WJ_3 | 99.41 | 220 | 281.42 | 3.02 | 6.74 |
| WB_1 | 99.41 | 240 | 345.29 | 3.33 | 9.28 |
| WB_2 | 99.31 | 293 | 386.84 | 4.00 | 22.32 |
| WB_3 | 99.35 | 304 | 373.26 | 4.19 | 34.18 |
| NI_1 | 99.75 | 109 | 172.00 | 2.67 | 7.55 |
| NI_2 | 99.68 | 115 | 154.38 | 2.65 | 7.27 |
| **Total** | 3775.92 | 9455 | 12523.95 | 133.15 | 630.95 |
| **Average** | 99.37 | 248.82 | 329.58 | 3.50 | 16.60 |

**Table S4.** Statistical analysis of alpha diversity indices across sampling sites at the genus level. (**a**) Global comparisons using Kruskal-Wallis H test (*p* < 0.05). (**b**) post-hoc pairwise comparisons using Dunn’s test with Benjamini-Hochberg correction (no pairwise comparisons reached the statistical significance threshold of *p_adj_* < 0.05).

**(a)**

| **Index** | **Kruskal-Wallis (*H*)** | ***p*-value** |
| --- | --- | --- |
| Chao1 | 8.61 | 0.13 |
| Shannon | 11.85 | 0.040 |
| Inverse Simpson | 11.94 | 0.040 |

**(b)**

| **Comparisons** | **Shannon** | **Shannon (*p_adj_*)** | **InvSimpson** | **InvSimpson (*p_adj_*)** |
| --- | --- | --- | --- | --- |
| Northernmost Island - Ny-Ålesund | -2.47 | 0.103 | -2.19 | 0.144 |
| Northernmost Island - Trinity Island | -1.51 | 0.282 | -1.08 | 0.525 |
| Northernmost Island - Wahlbergøya | -2.69 | 0.106 | -2.15 | 0.118 |
| Northernmost Island - Wahlenbergfjorden | -0.58 | 0.707 | 0.02 | 0.987 |
| Northernmost Island - Walrus Island | -2.13 | 0.124 | -1.8 | 0.179 |
| Ny-Ålesund - Trinity Island | 0.44 | 0.705 | 0.74 | 0.628 |
| Ny-Ålesund - Wahlbergøya | -1 | 0.434 | -0.54 | 0.682 |
| Ny-Ålesund - Wahlenbergfjorden | 2.1 | 0.106 | 2.64 | 0.125 |
| Ny-Ålesund - Walrus Island | 0.47 | 0.735 | 0.6 | 0.689 |
| Trinity Island - Wahlbergøya | -1.04 | 0.445 | -0.97 | 0.554 |
| Trinity Island - Wahlenbergfjorden | 1.08 | 0.47 | 1.2 | 0.494 |
| Trinity Island - Walrus Island | -0.19 | 0.853 | -0.41 | 0.733 |
| Wahlbergøya - Wahlenbergfjorden | 2.37 | 0.089 | 2.42 | 0.115 |
| Wahlbergøya - Walrus Island | 1.23 | 0.411 | 0.87 | 0.58 |
| Wahlenbergfjorden - Walrus Island | -1.71 | 0.218 | -2.14 | 0.097 |

**Table S5.** Average ± standard deviation of the top 20 most abundant bacterial genera across sites. Phylotypes that could not be identified the genus names were denoted by small letter to the deepest taxonomic names that could be identified (c_ for class; o_, order; f_, family; and g_, genus).

| **Bacterial Phyla** | **Bacterial Genera** | **Ny-Ålesund** | **Trinity Island** | **Walrus Island** | **Wahlen-bergfjorden** | **Wahl-bergøya** | **Northernmost Island** |
| --- | --- | --- | --- | --- | --- | --- | --- |
| **Acidobacteria** | f__Acidobacteriaceae | 0.41 ± 0.26 | 0.01 ± 0.00 | 0.04 ± 0.02 | 0.01 ± 0.01 | 0.00 ± 0.00 | 23.71 ± 3.48 |
| **Actinobacteria** | g__Ilumatobacter | 4.88 ± 1.16 | 3.71 ± 2.01 | 2.65 ± 0.84 | 3.40 ± 0.48 | 0.36 ± 0.16 | 0.00 ± 0.00 |
|  | f__C111 | 3.61 ± 0.76 | 2.58 ± 1.56 | 0.85 ± 0.21 | 2.09 ± 0.27 | 0.81 ± 0.34 | 0.00 ± 0.00 |
|  | f__JdFBGBact | 2.29 ± 0.58 | 1.56 ± 0.91 | 0.73 ± 0.28 | 1.96 ± 0.30 | 0.01 ± 0.00 | 0.00 ± 0.00 |
|  | o__Actinomycetales | 1.65 ± 0.33 | 2.08 ± 1.69 | 3.55 ± 1.25 | 0.20 ± 0.04 | 4.65 ± 0.78 | 2.66 ± 0.46 |
| **Bacteroidetes** | f__Flavobacteriaceae | 2.74 ± 0.84 | 1.21 ± 0.80 | 2.04 ± 0.67 | 1.62 ± 0.36 | 0.05 ± 0.00 | 0.01 ± 0.01 |
| **Proteobacteria** | f__Rhodobacteraceae | 12.31 ± 2.25 | 23.49 ± 4.20 | 9.95 ± 2.03 | 33.69 ± 1.46 | 1.14 ± 0.27 | 0.03 ± 0.01 |
|  | c__Alphaproteobacteria | 2.91 ± 0.43 | 1.61 ± 0.56 | 2.12 ± 0.56 | 1.23 ± 0.19 | 1.18 ± 0.11 | 2.31 ± 0.27 |
|  | o__Rhizobiales | 2.63 ± 0.77 | 0.53 ± 0.29 | 1.10 ± 0.36 | 0.47 ± 0.10 | 3.25 ± 0.52 | 2.14 ± 0.65 |
|  | o__Sphingomonadales | 2.62 ± 0.41 | 2.28 ± 0.82 | 0.85 ± 0.20 | 0.27 ± 0.04 | 1.76 ± 0.23 | 0.14 ± 0.11 |
|  | f__Acetobacteraceae | 0.47 ± 0.26 | 0.08 ± 0.06 | 0.22 ± 0.11 | 0.03 ± 0.01 | 1.50 ± 0.44 | 23.58 ± 2.69 |
|  | g__Loktanella | 1.64 ± 0.49 | 5.54 ± 0.41 | 0.52 ± 0.22 | 4.29 ± 0.45 | 0.03 ± 0.01 | 0.00 ± 0.00 |
|  | f__Sphingomonadaceae | 1.79 ± 0.58 | 0.42 ± 0.12 | 0.36 ± 0.11 | 0.03 ± 0.02 | 3.87 ± 0.39 | 0.97 ± 0.62 |
|  | g__Sulfitobacter | 1.16 ± 0.23 | 5.91 ± 1.34 | 0.81 ± 0.20 | 1.98 ± 0.03 | 0.01 ± 0.00 | 0.00 ± 0.00 |
|  | f__Comamonadaceae | 1.37 ± 0.44 | 2.16 ± 0.68 | 1.31 ± 0.37 | 0.04 ± 0.03 | 2.91 ± 0.34 | 0.71 ± 0.07 |
|  | g__Sulfurovum | 1.53 ± 0.80 | 0.03 ± 0.00 | 2.56 ± 1.38 | 1.62 ± 0.15 | 0.00 ± 0.00 | 0.00 ± 0.00 |
|  | f__Helicobacteraceae | 1.78 ± 0.92 | 0.02 ± 0.02 | 1.78 ± 0.84 | 2.08 ± 0.35 | 0.00 ± 0.00 | 0.00 ± 0.00 |
|  | c__Gammaproteobacteria | 5.58 ± 1.05 | 3.28 ± 1.11 | 10.66 ± 3.33 | 7.87 ± 0.52 | 2.00 ± 1.14 | 0.26 ± 0.06 |
|  | g__Glaciecola | 1.79 ± 0.67 | 0.19 ± 0.14 | 1.70 ± 0.71 | 0.05 ± 0.01 | 0.00 ± 0.00 | 0.00 ± 0.00 |
|  | g__Pseudomonas | 0.36 ± 0.20 | 0.41 ± 0.02 | 0.03 ± 0.01 | 0.00 ± 0.00 | 11.68 ± 9.18 | 0.01 ± 0.00 |

**Table S6.** Beta diversity statistical differences among (or between) selected microbiota based on ThetaYC dissimilarity metrics using (**a**) AMOVA, and (**b**) PERMANOVA without and with PERMDISP. Statistical differences are denoted as follows: *p* < 0.05 (*), *p* < 0.01 (**), and *p* < 0.001 (***).

**(a)**

| **Soil sites** | ***p*-value** |
| --- | --- |
| Ny-Ålesund vs. Trinity Island vs. Walrus Island vs.  Wahlenbergfjorden vs. Wahlbergøya vs. Northernmost Island | **< 0.001***** |
| Ny-Ålesund vs. Northernmost Island | **0.002**** |
| Trinity Island vs. Northernmost Island | **0.326** |
| Walrus Island vs. Northernmost Island | **0.01*** |
| Wahlenbergfjorden vs. Northernmost Island | **0.098** |
| Wahlbergøya vs. Northernmost Island | **0.106** |
| Ny-Ålesund vs Trinity Island | **0.319** |
| Ny-Ålesund vs Walrus Island | **0.356** |
| Ny-Ålesund vs Wahlenbergfjorden | **0.272** |
| Ny-Ålesund vs Wahlbergøya | **< 0.001***** |
| Trinity Island vs Walrus Island | **0.479** |
| Trinity Island vs Wahlenbergfjorden | **0.108** |
| Trinity Island vs Wahlbergøya | **0.105** |
| Walrus Island vs Wahlenbergfjorden | **0.604** |
| Walrus Island vs Wahlbergøya | **0.005**** |
| Wahlenbergfjorden vs Wahlbergøya | **0.11** |

**(b)**

| **Analysis** | **Sources** | ***Degree of freedom (n-1)*** | ***p*-value** |
| --- | --- | --- | --- |
| **PERMANOVA** | Groups | 5 | **0.001**** |
|  | Residuals | 32 |  |
| **PERMDISP** | Groups | 5 | **0.020*** |
|  | Residuals | 32 |  |

**Table S7.** ASV-based bacterial community compositions across Arctic soil sampling sites, showing as average relative abundance (± SD) of (**a**) dominant phyla and (**b**) genera.

**(a)**

| **Bacterial phyla** | **Ny-Ålesund** | **Trinity Island** | **Walrus Island** | **Wahlen-bergfjorden** | **Wahlbergøya** | **Northernmost Island** |
| --- | --- | --- | --- | --- | --- | --- |
| **Proteobacteria** | 52.99 ± 10.31 | 68.82 ± 22.13 | 50.21 ± 17.14 | 72.40 ± 3.96 | 54.21 ± 20.09 | 44.16 ± 1.04 |
| **Actinobacteriota** | 20.17 ± 9.41 | 17.55 ± 17.09 | 14.33 ± 8.43 | 10.12 ± 2.58 | 33.83 ± 16.63 | 7.72 ± 5.46 |
| **Bacteroidota** | 7.11 ± 9.34 | 4.09 ± 2.26 | 5.33 ± 4.40 | 3.36 ± 0.98 | 1.76 ± 1.45 | 0.00 ± 0.16 |
| **Acidobacteriota** | 3.56 ± 5.60 | 0.00 ± 0.01 | 2.41 ± 3.64 | 0.00 ± 0.12 | 2.64 ± 2.18 | 39.91 ± 2.89 |
| **Campilobacterota** | 3.50 ± 7.84 | 0.00 ± 0.15 | 4.54 ± 6.52 | 3.23 ± 0.75 | 0.00 ± 0.00 | 0.00 ± 0.00 |
| **Gemmatimonadota** | 1.20 ± 1.99 | 0.00 ± 0.15 | 4.60 ± 7.07 | 0.00 ± 0.13 | 1.15 ± 0.60 | 0.00 ± 0.48 |
| **Firmicutes** | 0.00 ± 1.31 | 4.86 ± 6.88 | 0.00 ± 1.14 | 0.00 ± 0.44 | 0.00 ± 0.60 | 0.00 ± 0.00 |
| **Chloroflexi** | 0.00 ± 0.99 | 0.00 ± 0.00 | 1.28 ± 1.41 | 0.00 ± 0.21 | 1.45 ± 0.89 | 0.00 ± 0.00 |
| **Verrucomicrobiota** | 0.00 ± 0.54 | 0.00 ± 0.01 | 0.00 ± 0.73 | 1.28 ± 0.35 | 0.00 ± 0.14 | 0.00 ± 0.00 |
| **Desulfobacterota** | 0.00 ± 0.62 | 0.00 ± 0.16 | 2.66 ± 3.89 | 2.12 ± 0.61 | 0.00 ± 0.00 | 0.00 ± 0.00 |
| **Unclassified** | 7.25 ± 5.42 | 3.68 ± 0.87 | 11.99 ± 6.01 | 5.23 ± 1.59 | 2.76 ± 0.55 | 5.95 ± 0.40 |
| **Other phyla (>1%)** | 4.22 ± 0.00 | 1.00 ± 0.43 | 2.65 ± 0.00 | 2.27 ± 0.12 | 2.20 ± 0.00 | 2.26 ± 0.14 |

**(b)**

| **Bacterial phyla** | **Bacterial genera** | **Ny-Ålesund** | **Trinity Island** | **Walrus Island** | **Wahlen-bergfjorden** | **Wahl-bergøya** | **Northernmost Island** |
| --- | --- | --- | --- | --- | --- | --- | --- |
| Acidobacteriota | Acidobacteriaceae_unclassified | 0.38 ± 1.01 | 0.00 ± 0.00 | 0.00 ± 0.00 | 0.00 ± 0.00 | 0.00 ± 0.00 | 21.37 ± 3.45 |
| Acidobacteriota | *Granulicella* | 0.11 ± 0.35 | 0.00 ± 0.00 | 0.00 ± 0.00 | 0.00 ± 0.00 | 0.00 ± 0.00 | 8.00 ± 3.12 |
|  | *Ilumatobacter* | 5.13 ± 5.84 | 5.29 ± 5.00 | 1.60 ± 1.72 | 4.29 ± 1.07 | 0.29 ± 0.51 | 0.00 ± 0.00 |
|  | Actinobacteria_unclassified | 0.98 ± 1.03 | 1.91 ± 1.46 | 2.95 ± 3.98 | 0.27 ± 0.24 | 4.58 ± 2.60 | 2.12 ± 1.72 |
|  | Micrococcaceae_unclassified | 0.13 ± 0.20 | 0.11 ± 0.16 | 0.00 ± 0.00 | 0.00 ± 0.00 | 8.59 ± 6.44 | 0.00 ± 0.00 |
| Bacteroidota | Flavobacteriaceae_unclassified | 3.70 ± 5.41 | 1.21 ± 1.07 | 2.81 ± 2.91 | 1.66 ± 0.22 | 0.08 ± 0.14 | 0.00 ± 0.00 |
| Campilobacterota | *Sulfurovum* | 2.91 ± 6.77 | 0.00 ± 0.00 | 2.53 ± 3.96 | 2.65 ± 0.53 | 0.00 ± 0.00 | 0.00 ± 0.00 |
| Proteobacteria | Acetobacteraceae_unclassified | 0.40 ± 0.87 | 0.00 ± 0.00 | 0.39 ± 0.67 | 0.00 ± 0.00 | 1.00 ± 0.27 | 19.18 ± 4.68 |
|  | Alphaproteobacteria_unclassified | 3.45 ± 2.10 | 2.42 ± 2.46 | 2.52 ± 2.34 | 1.53 ± 0.61 | 1.46 ± 0.51 | 3.14 ± 0.28 |
|  | Rhodobacteraceae_unclassified | 12.89 ± 10.51 | 26.85 ± 5.97 | 11.87 ± 7.96 | 29.92 ± 0.79 | 1.44 ± 1.11 | 0.00 ± 0.00 |
|  | *Yoonia-Loktanella* | 1.73 ± 2.57 | 3.02 ± 1.80 | 0.30 ± 0.66 | 6.88 ± 1.10 | 0.08 ± 0.14 | 0.00 ± 0.00 |
|  | *Sulfitobacter* | 0.35 ± 0.41 | 2.33 ± 0.73 | 0.29 ± 0.43 | 5.88 ± 4.60 | 0.00 ± 0.00 | 0.00 ± 0.00 |
|  | Sphingomonadaceae_unclassified | 3.35 ± 2.68 | 2.65 ± 1.83 | 1.14 ± 0.73 | 0.22 ± 0.21 | 8.75 ± 2.59 | 0.79 ± 0.80 |
|  | Comamonadaceae_unclassified | 1.46 ± 2.47 | 2.11 ± 0.42 | 1.19 ± 1.51 | 0.00 ± 0.00 | 2.35 ± 0.63 | 0.68 ± 0.64 |
|  | Gammaproteobacteria_unclassified | 4.13 ± 3.20 | 4.08 ± 2.89 | 9.89 ± 9.19 | 5.59 ± 1.90 | 1.93 ± 1.99 | 0.45 ± 0.31 |
|  | *Pseudomonas* | 0.29 ± 0.76 | 0.11 ± 0.15 | 0.00 ± 0.00 | 0.00 ± 0.00 | 7.52 ± 10.42 | 0.11 ± 0.16 |
|  | Pseudomonadaceae_unclassified | 0.30 ± 0.84 | 0.22 ± 0.01 | 0.10 ± 0.17 | 0.00 ± 0.00 | 5.77 ± 7.07 | 0.00 ± 0.00 |
|  | *Cocleimonas* | 0.54 ± 0.70 | 0.11 ± 0.16 | 1.66 ± 1.73 | 4.39 ± 2.02 | 0.00 ± 0.00 | 0.00 ± 0.00 |
|  | Other genera (<1%) | 45.04 ± 0.53 | 41.66 ± 0.44 | 43.06 ± 0.82 | 25.58 ± 0.62 | 51.64 ± 0.58 | 36.96 ± 0.94 |
|  | Unclassified genera | 12.75 ± 5.42 | 5.92 ± 0.87 | 17.71 ± 6.01 | 11.13 ± 1.59 | 4.51 ± 0.55 | 7.19 ± 0.40 |

**Table S8**. Predicted functional profiles of Arctic soil microbial communities using PICRUSt2. (**a**) Average relative abundance (± SD) of the top 30 predicted MetaCyc functional pathways. (**b**) Weighted nearest sequenced taxon index (NSTI) values for individual soil sample, representing the accuracy of its functional profile prediction.

**(a)**

| **MetaCyc pathways** | **Ny-Ålesund** | **Trinity Island** | **Walrus Island** | **Wahlen-bergfjorden** | **Wahl-bergøya** | **Northernmost Island** |
| --- | --- | --- | --- | --- | --- | --- |
| Aerobic respiration I (cytochrome c) | 1.68 ± 0.05 | 1.64 ± 0.05 | 1.71 ± 0.06 | 1.62 ± 0.01 | 1.56 ± 0.08 | 1.91 ± 0.03 |
| Pyruvate fermentation to isobutanol (engineered) | 1.09 ± 0.08 | 1.26 ± 0.01 | 1.03 ± 0.03 | 1.17 ± 0.01 | 1.09 ± 0.06 | 1.09 ± 0.04 |
| L-isoleucine biosynthesis II | 1.10 ± 0.09 | 1.31 ± 0.08 | 1.06 ± 0.06 | 1.26 ± 0.02 | 0.92 ± 0.06 | 1.01 ± 0.00 |
| L-valine biosynthesis | 1.06 ± 0.12 | 1.33 ± 0.09 | 1.02 ± 0.07 | 1.30 ± 0.03 | 0.87 ± 0.06 | 0.96 ± 0.01 |
| L-isoleucine biosynthesis I (from threonine) | 1.06 ± 0.12 | 1.33 ± 0.09 | 1.02 ± 0.07 | 1.30 ± 0.03 | 0.87 ± 0.06 | 0.96 ± 0.01 |
| Superpathway of branched amino acid biosynthesis | 0.93 ± 0.07 | 1.03 ± 0.02 | 0.91 ± 0.03 | 1.01 ± 0.01 | 0.77 ± 0.05 | 0.87 ± 0.02 |
| Gondoate biosynthesis (anaerobic) | 0.95 ± 0.06 | 1.05 ± 0.13 | 0.92 ± 0.10 | 1.09 ± 0.02 | 0.66 ± 0.13 | 0.91 ± 0.00 |
| Fatty acid elongation -- saturated | 0.91 ± 0.07 | 1.05 ± 0.13 | 0.85 ± 0.10 | 1.05 ± 0.02 | 0.64 ± 0.08 | 0.88 ± 0.00 |
| Cis-vaccenate biosynthesis | 0.81 ± 0.04 | 0.81 ± 0.06 | 0.82 ± 0.04 | 0.87 ± 0.02 | 0.67 ± 0.10 | 0.91 ± 0.00 |
| L-isoleucine biosynthesis III | 0.82 ± 0.04 | 0.86 ± 0.01 | 0.82 ± 0.02 | 0.86 ± 0.00 | 0.72 ± 0.03 | 0.80 ± 0.01 |
| Superpathway of phospholipid biosynthesis I (bacteria) | 0.74 ± 0.04 | 0.74 ± 0.04 | 0.74 ± 0.03 | 0.74 ± 0.01 | 0.68 ± 0.02 | 0.78 ± 0.01 |
| TCA cycle I (prokaryotic) | 0.77 ± 0.03 | 0.75 ± 0.03 | 0.79 ± 0.02 | 0.77 ± 0.00 | 0.66 ± 0.04 | 0.79 ± 0.02 |
| CDP-diacylglycerol biosynthesis I | 0.77 ± 0.03 | 0.78 ± 0.00 | 0.77 ± 0.02 | 0.78 ± 0.00 | 0.70 ± 0.02 | 0.83 ± 0.00 |
| CDP-diacylglycerol biosynthesis II | 0.77 ± 0.03 | 0.78 ± 0.00 | 0.77 ± 0.02 | 0.78 ± 0.00 | 0.70 ± 0.02 | 0.83 ± 0.00 |
| Superpathway of L-isoleucine biosynthesis I | 0.69 ± 0.03 | 0.69 ± 0.01 | 0.70 ± 0.02 | 0.70 ± 0.00 | 0.63 ± 0.03 | 0.76 ± 0.01 |
| Superpathway of adenosine nucleotides de novo biosynthesis II | 0.70 ± 0.03 | 0.69 ± 0.00 | 0.72 ± 0.02 | 0.69 ± 0.00 | 0.65 ± 0.03 | 0.73 ± 0.02 |
| Superpathway of adenosine nucleotides de novo biosynthesis I | 0.72 ± 0.03 | 0.72 ± 0.00 | 0.74 ± 0.02 | 0.72 ± 0.00 | 0.67 ± 0.03 | 0.76 ± 0.02 |
| Adenosine ribonucleotides de novo biosynthesis | 0.72 ± 0.03 | 0.72 ± 0.00 | 0.74 ± 0.03 | 0.72 ± 0.00 | 0.67 ± 0.04 | 0.72 ± 0.02 |
| TCA cycle V (2-oxoglutarate:ferredoxin oxidoreductase) | 0.73 ± 0.03 | 0.65 ± 0.08 | 0.75 ± 0.03 | 0.70 ± 0.01 | 0.63 ± 0.05 | 0.74 ± 0.03 |
| Phosphatidylglycerol biosynthesis II (non-plastidic) | 0.71 ± 0.04 | 0.70 ± 0.03 | 0.71 ± 0.03 | 0.71 ± 0.01 | 0.65 ± 0.02 | 0.74 ± 0.00 |
| Phosphatidylglycerol biosynthesis I (plastidic) | 0.71 ± 0.04 | 0.70 ± 0.03 | 0.71 ± 0.03 | 0.71 ± 0.01 | 0.65 ± 0.02 | 0.74 ± 0.00 |
| Superpathway of L-serine and glycine biosynthesis I | 0.66 ± 0.06 | 0.72 ± 0.01 | 0.64 ± 0.07 | 0.70 ± 0.00 | 0.62 ± 0.04 | 0.69 ± 0.00 |
| Adenosine deoxyribonucleotides de novo biosynthesis II | 0.67 ± 0.03 | 0.65 ± 0.00 | 0.69 ± 0.02 | 0.65 ± 0.00 | 0.62 ± 0.02 | 0.75 ± 0.01 |
| Guanosine deoxyribonucleotides de novo biosynthesis II | 0.67 ± 0.03 | 0.65 ± 0.00 | 0.69 ± 0.02 | 0.65 ± 0.00 | 0.62 ± 0.02 | 0.75 ± 0.01 |
| 5-Aminoimidazole ribonucleotide biosynthesis II | 0.69 ± 0.04 | 0.66 ± 0.02 | 0.69 ± 0.04 | 0.65 ± 0.00 | 0.63 ± 0.04 | 0.72 ± 0.01 |
| Superpathway of 5-aminoimidazole ribonucleotide biosynthesis | 0.69 ± 0.04 | 0.66 ± 0.02 | 0.69 ± 0.04 | 0.65 ± 0.00 | 0.63 ± 0.04 | 0.72 ± 0.01 |
| Superpathway of pyrimidine ribonucleotides de novo biosynthesis | 0.68 ± 0.04 | 0.65 ± 0.01 | 0.70 ± 0.03 | 0.66 ± 0.00 | 0.62 ± 0.02 | 0.73 ± 0.02 |
| UMP biosynthesis | 0.68 ± 0.03 | 0.65 ± 0.01 | 0.70 ± 0.03 | 0.66 ± 0.00 | 0.62 ± 0.02 | 0.73 ± 0.01 |
| Fatty acid salvage | 0.75 ± 0.12 | 0.78 ± 0.09 | 0.66 ± 0.10 | 0.65 ± 0.01 | 0.74 ± 0.04 | 0.54 ± 0.05 |
| Pentose phosphate pathway (non-oxidative branch) | 0.67 ± 0.07 | 0.64 ± 0.00 | 0.69 ± 0.08 | 0.59 ± 0.01 | 0.77 ± 0.03 | 0.65 ± 0.02 |

**(b)**

| **Sample ID** | **Weighted NSTI** |
| --- | --- |
| NA1_1 | 0.4299 |
| NA1_2 | 0.4324 |
| NA1_3 | 0.4284 |
| NA2_1 | 0.3113 |
| NA2_2 | 0.2669 |
| NA2_3 | 0.2915 |
| NA3_1 | 0.3921 |
| NA3_2 | 0.3896 |
| NA3_3 | 0.3941 |
| NA4_1 | 0.7193 |
| NA4_2 | 0.7221 |
| NA4_3 | 0.8282 |
| NA5_1 | 0.2764 |
| NA5_2 | 0.3050 |
| NA5_3 | 0.3002 |
| NA6_1 | 0.3740 |
| NA6_2 | 0.3808 |
| NA6_3 | 0.3876 |
| TI_1 | 0.3018 |
| TI_2 | 0.2151 |
| WI1_1 | 0.4381 |
| WI1_2 | 0.4498 |
| WI1_3 | 0.5278 |
| WI2_1 | 0.6994 |
| WI2_2 | 0.7330 |
| WI2_3 | 0.8685 |
| WI3_1 | 0.4584 |
| WI3_2 | 0.3963 |
| WI4_1 | 0.5072 |
| WI4_2 | 0.4718 |
| WJ_1 | 0.3102 |
| WJ_2 | 0.2757 |
| WJ_3 | 0.2672 |
| WB_1 | 0.3279 |
| WB_2 | 0.3541 |
| WB_3 | 0.3821 |
| NI_1 | 0.3956 |
| NI_2 | 0.3272 |

**Table S9.** (**a**) Species classification of core phylotypes (phylotypes that could not be identified the species names were denoted by small letter to the deepest taxonomic names that could be identified (c_ for class; o_, order; f_, family; g_, genus; and s, species)) and (**b**) SparCC correlation values and types, depicted in **Fig. 6b**. In (**b**), only statistically significant correlations are presented (|ρ| > 0.8, FDR-adjusted *p* < 0.01).

**(a)**

| Phylo ID | Phylum | Class | Species |
| --- | --- | --- | --- |
| Phylo0001 | Proteobacteria | Alphaproteobacteria | f__Rhodobacteraceae |
| Phylo0003 | Proteobacteria | Gammaproteobacteria | c__Gammaproteobacteria |
| Phylo0004 | Actinobacteria | Acidimicrobiia | s__Ilumatobacter_fluminis |
| Phylo0005 | Actinobacteria | Acidimicrobiia | f__C111 |
| Phylo0006 | Proteobacteria | Alphaproteobacteria | c__Alphaproteobacteria |
| Phylo0009 | Actinobacteria | Actinobacteria | o__Actinomycetales |
| Phylo0010 | Proteobacteria | Alphaproteobacteria | o__Sphingomonadales |
| Phylo0011 | Proteobacteria | Alphaproteobacteria | o__Rhizobiales |
| Phylo0012 | Bacteroidetes | Flavobacteriia | f__Flavobacteriaceae |
| Phylo0013 | Proteobacteria | Epsilonproteobacteria | f__Helicobacteraceae |
| Phylo0014 | Proteobacteria | Alphaproteobacteria | f__Acetobacteraceae |
| Phylo0015 | Actinobacteria | Acidimicrobiia | f__JdFBGBact |
| Phylo0016 | Acidobacteria | Acidobacteriia | f__Acidobacteriaceae |
| Phylo0017 | Proteobacteria | Epsilonproteobacteria | s__Sulfurovum_lithotrophicum |
| Phylo0018 | Proteobacteria | Alphaproteobacteria | f__Sphingomonadaceae |
| Phylo0019 | Proteobacteria | Betaproteobacteria | f__Comamonadaceae |
| Phylo0020 | Proteobacteria | Alphaproteobacteria | g__Loktanella |
| Phylo0021 | Actinobacteria | Actinobacteria | g__Arthrobacter |
| Phylo0022 | Proteobacteria | Gammaproteobacteria | g__Cocleimonas |
| Phylo0023 | Proteobacteria | Deltaproteobacteria | f__Desulfobulbaceae |
| Phylo0024 | Proteobacteria | Alphaproteobacteria | g__Sulfitobacter |
| Phylo0026 | Proteobacteria | Alphaproteobacteria | g__Rhodobacter |
| Phylo0029 | Proteobacteria | Gammaproteobacteria | f__[Marinicellaceae] |
| Phylo0031 | Proteobacteria | Alphaproteobacteria | s__Loktanella_salsilacus |
| Phylo0032 | Acidobacteria | Solibacteres | o__Solibacterales |
| Phylo0036 | Actinobacteria | Actinobacteria | g__Nocardioides |
| Phylo0037 | Proteobacteria | Alphaproteobacteria | g__Kaistobacter |
| Phylo0039 | Proteobacteria | Alphaproteobacteria | s__Octadecabacter_antarcticus |
| Phylo0040 | Proteobacteria | Alphaproteobacteria | f__Caulobacteraceae |
| Phylo0041 | Actinobacteria | Acidimicrobiia | f__koll13 |
| Phylo0042 | Acidobacteria | Acidobacteriia | g__Granulicella |
| Phylo0050 | Proteobacteria | Alphaproteobacteria | g__Sphingomonas |
| Phylo0053 | Actinobacteria | Actinobacteria | f__Microbacteriaceae |
| Phylo0056 | Actinobacteria | Actinobacteria | f__Intrasporangiaceae |
| Phylo0059 | Proteobacteria | Gammaproteobacteria | g__Psychromonas |
| Phylo0061 | Proteobacteria | Alphaproteobacteria | g__Zymomonas |
| Phylo0065 | Proteobacteria | Gammaproteobacteria | g__Rhodanobacter |
| Phylo0066 | Proteobacteria | Betaproteobacteria | f__Oxalobacteraceae |
| Phylo0069 | Proteobacteria | Alphaproteobacteria | s__Sulfitobacter_guttiformis |
| Phylo0070 | Proteobacteria | Alphaproteobacteria | g__Devosia |
| Phylo0073 | Proteobacteria | Gammaproteobacteria | g__Leucothrix |
| Phylo0075 | Proteobacteria | Gammaproteobacteria | s__Psychromonas_japonica |
| Phylo0080 | Proteobacteria | Gammaproteobacteria | g__Psychrobacter |
| Phylo0086 | Proteobacteria | Alphaproteobacteria | f__Rhizobiaceae |
| Phylo0106 | Proteobacteria | Gammaproteobacteria | g__Shewanella |
| Phylo0136 | Actinobacteria | Actinobacteria | f__Thermomonosporaceae |
| Phylo0137 | Armatimonadetes | Armatimonadia | o__FW68 |
| Phylo0148 | Acidobacteria | Acidobacteriia | s__Granulicella_aggregans |

**(b)**

| **Source** | **Target** | **Correlation** | **Correlation Type** |
| --- | --- | --- | --- |
| Phylo0001 | Phylo0032 | -0.86159 | Negative |
| Phylo0001 | Phylo0015 | 0.802342 | Positive |
| Phylo0001 | Phylo0029 | 0.804879 | Positive |
| Phylo0001 | Phylo0004 | 0.85308 | Positive |
| Phylo0001 | Phylo0024 | 0.877507 | Positive |
| Phylo0003 | Phylo0014 | -0.84452 | Negative |
| Phylo0003 | Phylo0032 | -0.83235 | Negative |
| Phylo0003 | Phylo0011 | -0.81983 | Negative |
| Phylo0003 | Phylo0015 | 0.834214 | Positive |
| Phylo0003 | Phylo0022 | 0.877539 | Positive |
| Phylo0003 | Phylo0012 | 0.904869 | Positive |
| Phylo0004 | Phylo0014 | -0.84453 | Negative |
| Phylo0004 | Phylo0032 | -0.84373 | Negative |
| Phylo0004 | Phylo0015 | 0.81089 | Positive |
| Phylo0004 | Phylo0005 | 0.86844 | Positive |
| Phylo0009 | Phylo0022 | -0.82524 | Negative |
| Phylo0009 | Phylo0039 | -0.80411 | Negative |
| Phylo0011 | Phylo0012 | -0.92437 | Negative |
| Phylo0011 | Phylo0022 | -0.84526 | Negative |
| Phylo0011 | Phylo0039 | -0.80051 | Negative |
| Phylo0012 | Phylo0032 | -0.8633 | Negative |
| Phylo0012 | Phylo0024 | 0.843862 | Positive |
| Phylo0012 | Phylo0039 | 0.862923 | Positive |
| Phylo0012 | Phylo0015 | 0.896568 | Positive |
| Phylo0012 | Phylo0022 | 0.898649 | Positive |
| Phylo0013 | Phylo0017 | 0.966441 | Positive |
| Phylo0014 | Phylo0032 | 0.866859 | Positive |
| Phylo0014 | Phylo0040 | 0.878585 | Positive |
| Phylo0015 | Phylo0032 | -0.85973 | Negative |
| Phylo0015 | Phylo0050 | -0.82034 | Negative |
| Phylo0015 | Phylo0022 | 0.848918 | Positive |
| Phylo0015 | Phylo0024 | 0.873873 | Positive |
| Phylo0015 | Phylo0039 | 0.9115 | Positive |
| Phylo0016 | Phylo0137 | 0.806919 | Positive |
| Phylo0016 | Phylo0148 | 0.811728 | Positive |
| Phylo0016 | Phylo0065 | 0.830789 | Positive |
| Phylo0016 | Phylo0042 | 0.861216 | Positive |
| Phylo0017 | Phylo0023 | 0.806289 | Positive |
| Phylo0020 | Phylo0041 | 0.814357 | Positive |
| Phylo0020 | Phylo0029 | 0.855596 | Positive |
| Phylo0021 | Phylo0037 | 0.84142 | Positive |
| Phylo0021 | Phylo0066 | 0.864007 | Positive |
| Phylo0022 | Phylo0032 | -0.80814 | Negative |
| Phylo0022 | Phylo0024 | 0.819095 | Positive |
| Phylo0022 | Phylo0039 | 0.844403 | Positive |
| Phylo0024 | Phylo0032 | -0.88166 | Negative |
| Phylo0024 | Phylo0039 | 0.879043 | Positive |
| Phylo0032 | Phylo0040 | 0.833649 | Positive |
| Phylo0032 | Phylo0050 | 0.846201 | Positive |
| Phylo0037 | Phylo0086 | 0.804721 | Positive |
| Phylo0037 | Phylo0066 | 0.837507 | Positive |
| Phylo0040 | Phylo0050 | 0.861953 | Positive |
| Phylo0042 | Phylo0137 | 0.857377 | Positive |
| Phylo0042 | Phylo0148 | 0.878582 | Positive |
| Phylo0042 | Phylo0065 | 0.879855 | Positive |
| Phylo0042 | Phylo0136 | 0.887392 | Positive |
| Phylo0065 | Phylo0137 | 0.819502 | Positive |
| Phylo0065 | Phylo0136 | 0.83729 | Positive |
| Phylo0065 | Phylo0148 | 0.84766 | Positive |
| Phylo0136 | Phylo0137 | 0.808569 | Positive |
| Phylo0136 | Phylo0148 | 0.8436 | Positive |
| Phylo0137 | Phylo0148 | 0.831056 | Positive |
